# Population Genomics Reveals Incipient Speciation, Introgression, and Adaptation in the African Mona Monkey (*Cercopithecus mona*)

**DOI:** 10.1093/molbev/msaa248

**Published:** 2020-09-28

**Authors:** Adeola Oluwakemi Ayoola, Bao-Lin Zhang, Richard P Meisel, Lotanna M Nneji, Yong Shao, Olanrewaju B Morenikeji, Adeniyi C Adeola, Said I Ng’ang’a, Babafemi G Ogunjemite, Agboola O Okeyoyin, Christian Roos, Dong-Dong Wu

**Affiliations:** 1 State Key Laboratory of Genetic Resources and Evolution, Kunming Institute of Zoology, Chinese Academy of Sciences, Kunming, Yunnan, China; 2 Kunming College of Life Science, University of the Chinese Academy of Sciences, Kunming, Yunnan, China; 3 Department of Biology and Biochemistry, University of Houston, Houston, TX; 4 Sino-Africa Joint Research Center, Chinese Academy of Sciences, Kunming, Yunnan, China; 5 Department of Biomedical Sciences, Rochester Institute of Technology, Rochester, NY; 6 Department of Biology, Hamilton College, Clinton, NY; 7 Department of Ecotourism and Wildlife Management, Federal University of Technology, Akure, Nigeria; 8 National Park Service Headquarters, Federal Capital Territory, Abuja, Nigeria; 9 Gene Bank of Primates and Primate Genetics Laboratory, German Primate Center, Leibniz Institute for Primate Research, Göttingen, Germany; 10 National Resource Center for Non-Human Primates, Kunming Primate Research Center, and National Research Facility for Phenotypic & Genetic Analysis of Model Animals (Primate Facility), Kunming Institute of Zoology, Chinese Academy of Sciences, Kunming, Yunnan, China; 11 Center for Excellence in Animal Evolution and Genetics, Chinese Academy of Sciences, Kunming, Yunnan, China

**Keywords:** guenons, genome sequencing, incipient speciation, introgression, natural selection

## Abstract

Guenons (tribe *Cercopithecini*) are the most widely distributed nonhuman primate in the tropical forest belt of Africa and show considerable phenotypic, taxonomic, and ecological diversity. However, genomic information for most species within this group is still lacking. Here, we present a high-quality de novo genome (total 2.90 Gb, contig N50 equal to 22.7 Mb) of the mona monkey (*Cercopithecus mona*), together with genome resequencing data of 13 individuals sampled across Nigeria. Our results showed differentiation between populations from East and West of the Niger River ∼84 ka and potential ancient introgression in the East population from other mona group species. The *PTPRK*, *FRAS1*, *BNC2*, and *EDN3* genes related to pigmentation displayed signals of introgression in the East population. Genomic scans suggest that immunity genes such as *AKT3* and *IL13* (possibly involved in simian immunodeficiency virus defense), and *G6PD*, a gene involved in malaria resistance, are under positive natural selection. Our study gives insights into differentiation, natural selection, and introgression in guenons.

## Introduction

African nonhuman primates are exceptionally diverse, yet knowledge on their genetics, behavior, and ecology is largely limited, which has hindered their conservation and utilization as human disease models. In Africa, guenons (tribe *Cercopithecini*) are one of the most diverse groups of nonhuman primates and show great variation in ecology, behavior, and morphology. Several factors have been illustrated as potential drivers in the evolutionary radiation of guenons (Tosi et al. 2004, 2005). For instance, some guenons are terrestrial, whereas the majorities are arboreal forest-dwellers utilizing different forest canopy levels in sub-Saharan Africa (Gautier et al. 2002). Thus, previous studies (Disotell and Raaum 2002; Guschanski et al. 2013) have stated that speciation in guenons may be associated with forest cover change and specifically forest refugia that occurred in the last 10 Ma. Further, hybridization has also been identified as an important mechanism in the evolution of many lineages, including guenons (Allen et al. 2014; Detwiler 2019; van der Valk et al. 2020), which could have resulted in genetic introgression across species.

Among the five genera of this tribe (i.e., *Cercopithecus*, *Miopithecus*, *Allenopithecus*, *Erythrocebus*, and *Chlorocebus*), the genus *Cercopithecu*s is the largest, consisting of 26 species that exhibit variation in genetics, morphology, ecology, behavior, and social organization (Grubb et al. 2003; Anandam et al. 2013; Zinner et al. 2013). Previous studies based on mitochondrial and nuclear sequences (Tosi et al. 2006) have illustrated their phylogeography and identified several species groups, including the *dryas*, *diana*, *mitis*, *cephus*, *hamlyni*, *neglectus*, and *mona* groups (Grubb et al. 2003; Chatterjee et al. 2009; Guschanski et al. 2013). The mona species group has attracted growing attention (Oates 2011; Okekedunu et al. 2014; Onadeko et al. 2014) and is composed of several species or subspecies. Similarly, this group is widely distributed across the African Guineo-Congolian rainforest, with a range extending across Ghana to Cameroon (Oates 2011; Okekedunu et al. 2014). Mona group species live in several types of forest areas within urban, peri-urban, and wild populations (Okekedunu et al. 2014). They usually coexist in sympatry with other primates and often form polyspecific troops, allowing interspecific or even intergeneric hybridization (Gartlan and Struhsaker 1972; Goodwin 2007). Thus, this group provides an ideal model system to investigate the role of genetic introgression in primate evolution. Thus far, no genomic information has been reported for the mona group, which has made it difficult to delineate the evolutionary history and adaptive evolution of this species group.

The advent of large-scale genome sequencing has facilitated investigations into the evolution and ecology of many wild animals, particularly nonhuman primates (Gibbs et al. 2007; Locke et al. 2011; Scally et al. 2012; Prado-Martinez et al. 2013; Carbone et al. 2014; Rogers and Gibbs 2014; Worley et al. 2014; Rogers et al. 2019; Zhang et al. 2019; van der Valk et al. 2020). Herein, we de novo assembled the genome of *Cercopithecus mona* and utilized large-scale genomic data to investigate population differentiation, demographic history, natural selection, and genetic introgression in this species. Interestingly, population genomic analyses revealed a divergence between the East and West Central (WC) populations ∼84  ka and identified several immunity genes under positive natural selection. We also found some genes related to pigmentation displaying signals of genetic introgression in the East population from an unknown species. This study should help improve our understanding of the evolution and genetics of the African mona species group.

## Results

### De Novo Assembly of *C. mona* Genome by Nanopore Sequencing

**Fig. 1.**
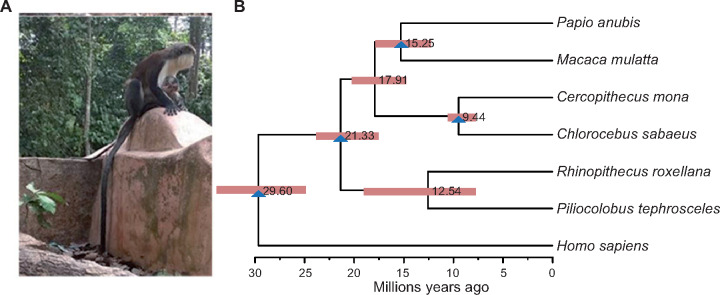
(*A*) Image of typical *Cercopithecus mona* taken at Osun-Osogbo Sacred Grove protected area. (*B*) Comparative genomics analysis among *C. mona* and six related primates. Node bars refer to 95% confidence intervals of divergence time and blue triangles indicate fossil-based calibration points (see Materials and Methods).

We sequenced the genome of a female *C. mona* individual using the long-read sequencing platform Oxford Nanopore PromethION. In total, ∼156-Gb Nanopore reads and ∼151-Gb Illumina Hiseq reads for correcting sequencing errors were generated. The assembled genome was 2.90 Gb with a contig N50 equal to 22.7 Mb (supplementary table S1, Supplementary Material online). The completeness score evaluated by Benchmarking Universal Single-Copy Orthologs was 93.2% (supplementary table S2, Supplementary Material online), indicating that the genome sequence showed high quality and continuity for downstream analyses. Transposable elements (TE) occupied 41.3% of the mona genome, 7.5% of which were long terminal repeats (supplementary table S3, Supplementary Material online). Gene prediction from multiple approaches identified 23,408 protein-coding genes, which is within the range identified in other primate assemblies in the NCBI database. Of these, we identified 20,826 gene families, 8,574 of which were single copies across seven closely related primates (supplementary fig. S1, Supplementary Material online). Estimation of the divergence time based on single-copy gene families suggested that *C. mona* diverged from *Chlorocebus sabaeus* ∼ 9.44 Ma (95% confidence interval [CI] = 7.7–10.5 Ma, fig. 1). This time-frame is similar to that estimated by Pozzi et al. (2014) (∼9.03 Ma) but slightly older than other estimates (e.g., Perelman et al. 2011; Guschanski et al. 2013), which may be the result of different data sets and analytical approaches. As comparative genomics analyses with other primate species to understand long-term evolution are outside the scope of this study, we only focused on within species variation to reveal recent evolutionary history of *C. mona* in the following sections.

### Population Structure

**Fig. 2.**
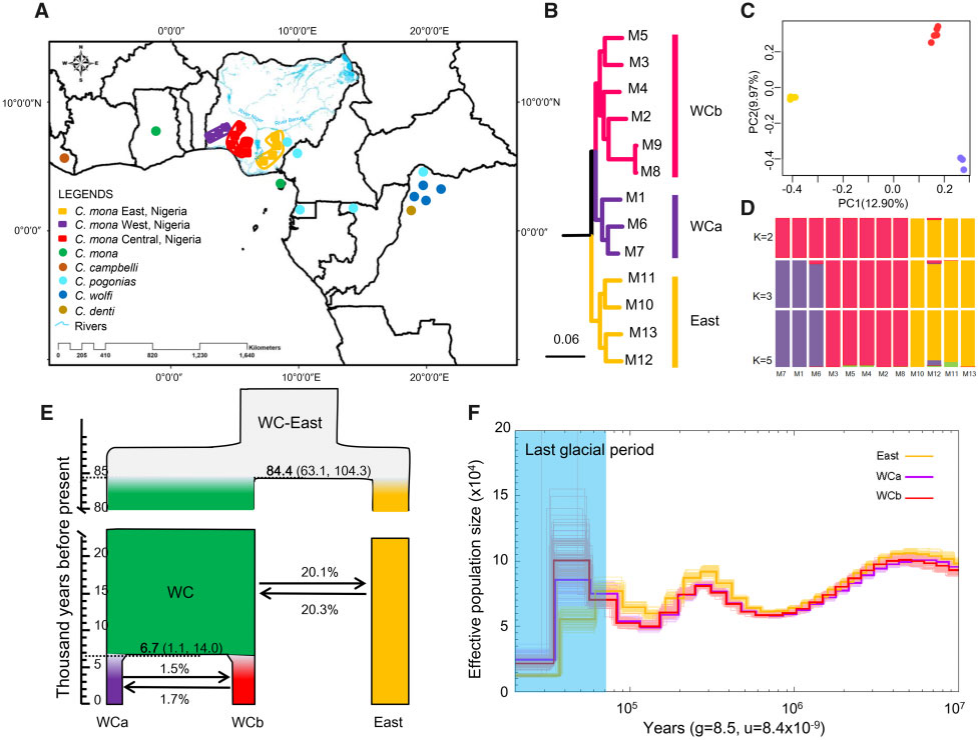
Population structure and demographic history of *Cercopithecus mona*. (*A*) Sample locations. Rectangles represent sample sites of *C. mona* used for genome resequencing in this study, and their colors correspond to clades recovered from phylogenetic and population structure analyses. Circle dots refer to mitochondrial genome sequences of other species downloaded from NCBI (supplementary table S10, Supplementary Material online). The geographic locations were described in the original study (Guschanski et al. 2013). (*B*) Maximum-likelihood tree of *C. mona* based on all concatenated SNPs. WCa (purple), WCb (red), and East (yellow) indicate three clades referring to West Central clades a and b and East clade, respectively. Outgroup is *Cercopithecus mitis* (not shown). (*C*) PCA results. (*D*) STRUCTURE analyses with *K* = 2–5. (*E*) Demographic history inferred from G-PhoCS results. Widths of branches are proportional to effective population size (Ne). Horizontal dashed lines denote posterior estimates for divergence times in thousand years before present, associated mean values are shown in bold, and 95% confidence intervals are given in parentheses. Arrows and numbers indicate direction and percentage of gene flow. (*F*) PSMC analyses (using a generation time of 8.5 years and an autosomal mutation rate of 8.415 × 10^−9^ per base pair per generation) show dynamic changes in effective population size. Last Glacial Period (20–70 ka) is shaded in light blue.

To investigate the population structure of *C. mona* in Africa, we sequenced the genomes of 13 additional *C. mona* individuals sampled across different coordinates in three geographical regions (East, Central, and West) of Nigeria and one *Cercopithecus mitis* individual as the outgroup (fig. 2*A*). These individuals were sequenced to an average depth of 24.5-fold (from 22 to 31) (supplementary table S4, Supplementary Material online). After mapping to the reference genome and filtering low-quality variants using the BWA-GATK programs (see Materials and Methods), we obtained 46.6 million high-quality single-nucleotide polymorphisms (SNPs) for downstream analyses. The phylogenetic tree based on all concatenated SNPs suggests that the *C. mona* individuals were composed of two distinct groups: that is, East and WC groups, separated by the Niger River (fig. 2*A* and *B*, bootstrap value = 100). The WC lineage could be further subdivided into WCa and WCb lineages without a clear geographic barrier. This topology was supported by trees inferred from coalescent methods (STAR) (Liu et al. 2009) and ASTRAL (Mirarab et al. 2014) based on 10,000 random neutral regions (supplementary fig. S2, Supplementary Material online). Similarly, the principal component analysis (PCA) separated the East and WC groups along the first eigenvector, which explained 12.90% of total genetic variance (fig. 2*C*), with the second eigenvector identifying WCa and WCb explaining 9.97% of the variance. The optimal number of genetic clusters inferred by STRUCTURE analysis was 3 (*K* = 3; supplementary fig. S3, Supplementary Material online) using 58,596 unlinked SNPs (fig. 2*D*).

### Demographic History and Population Differentiation

We first used the generalized phylogenetic coalescent sampler (G-PhoCS) program (Gronau et al. 2011) to infer divergence times, ancestral effective population sizes, and gene flow rates among the East, WCa, and WCb lineages. Based on comparison of the six models (supplementary fig. S4, Supplementary Material online), Model 3 best fitted the data (Akaike Information Criterion [AIC] = 2,732.30, supplementary tables S5–S7, Supplementary Material online), in which gene flow occurred between WCa and WCb, as well as between the East lineage and the ancestor of the WC population. We further used *D*-statistic analyses to evaluate genetic introgression and found no significant signal of admixture between the East and WCa or WCb samples (*Z* score < |3|, supplementary fig. S5 and table S8, Supplementary Material online). This suggests ancient gene flow between the East population and an ancestor of the WC population but limited recent gene flow between the East and WC populations after divergence between the WCa and WCb lineages. The total migration rate of gene flow from East to WC was 20.3%, which was nearly the same as the opposite direction (20.1%) (fig. 2*E*). The divergence time estimated from the best-fit model showed that the East lineage separated from the WC lineage ∼84.4 ka (95% CI = 63.1–104.3 ka), and the divergence between the two WC lineages occurred ∼6.7 ka (95% CI = 1.1–14.0 ka) (fig. 2*E* and supplementary table S6, Supplementary Material online).

Pairwise Sequentially Markovian Coalescent (PSMC) analyses showed a similar population size change trajectory for all lineages until ∼70 ka (fig. 2*F*). Thereafter, the East lineage experienced a great and prolonged decline in effective population size, whereas the WC lineages expanded 30–40% at first and then experienced a great decline ∼30 ka. Demographic history inferred from the Multiple Sequentially Markovian Coalescent (MSMC2) method showed a similar pattern as that of PSMC but exhibited population expansion for all lineages in recent years (<20 ka, supplementary fig. S6, Supplementary Material online). Considering that MSMC2 is more accurate than PSMC when reconstructing recent history, we suspect the population expansions in recent years by MSMC2 may be more reliable. In addition, the separation time estimated by MSMC2 between the East and WC linages was roughly 40–90 ka (supplementary fig. S7, Supplementary Material online), similar to the G-PhoCS results (fig. 2*E*).

### Rapid Evolution of Immunity Genes

**Fig. 3.**
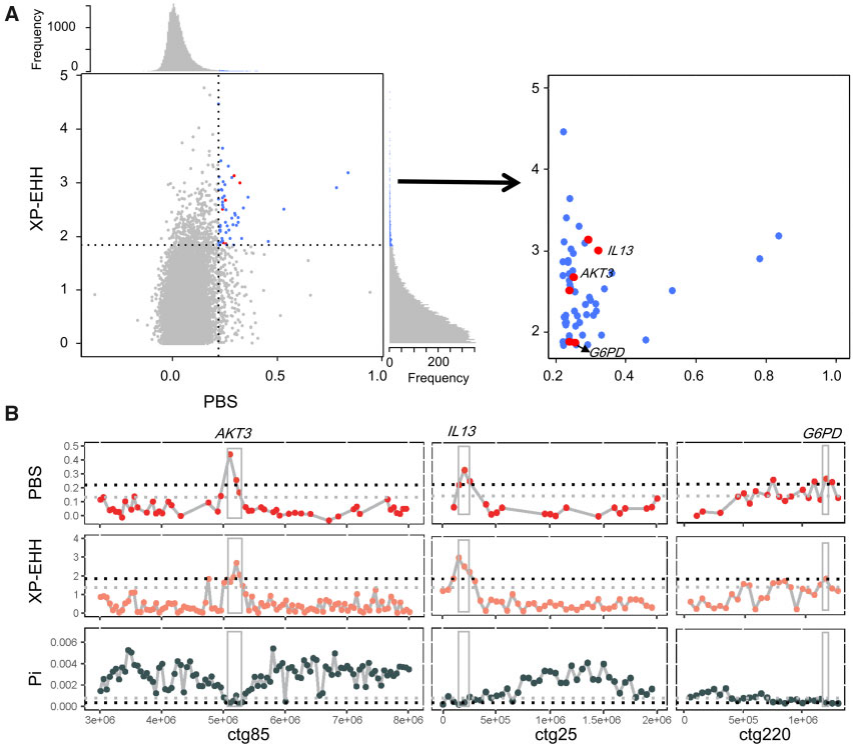
Selected genes in *Cercopithecus mona* East lineage. (*A*) Scatter plots of XP-EHH against PBS. Data points in blue correspond to genomic windows in top 1% PBS (=0.2190) and top 1% XP-EHH (=1.8369) ratio distribution. Windows also in low 1% Pi ratio distribution are marked in red, which represent candidate selective regions. Genes involved in SIV and malaria resistances are given. (*B*) Genes with strong selective sweep signals in *C. mona* East lineage. Gray and black dashed lines in PBS and XP-EHH indicate top 5% and 1% ratio distributions, respectively, whereas in Pi indicate lower 5% and 1% ratio distributions, respectively.

To identify genes showing rapid evolution, we first calculated genetic differentiation between the East and WC populations based on the population branch statistic (PBS) approach (see Materials and Methods). We focused on windows with extremely high divergence (top 1% PBS value). As a result, we identified 211 regions with a total length of 12.75 Mb, with the longest fragment being 300 kb and the shortest being 50 kb. From these highly divergent regions, 174 protein-coding genes were identified. Gene enrichment analysis indicated that many immunity pathways were significantly enriched (*P *<* *0.05, supplementary table S9, Supplementary Material online), for example, Toll-like genes that encode transmembrane proteins, and which recognize unique pathogen-associated molecular structures and play an important role in adaptive immune responses (Descamps et al. 2012). To validate the signal of positive selection on these genes, we performed further analyses to calculate Pi (nucleotide diversity) and cross-population extended haplotype homozygosity (XP-EHH). We identified ten protein-coding genes that showed extremely high PBS and XP-EHH values (top 1%) and extremely low Pi values (low 1%) in the East population compared with the WC population. Among these ten genes, we identified two genes related to immunity against simian immunodeficiency virus (SIV) infection: that is, AKT serine/threonine kinase 3 (*AKT3*) and interleukin 13 (*IL13*) (fig. 3). Moreover, we found that *G6PD* (encoding glucose-6-phosphate dehydrogenase), a well-known gene related to malaria infection, evolved under positive selection in the East population (fig. 3). Similarly, evolutionary studies suggest that recent selection has operated on *G6PD*-deficient alleles in modern humans, thought to be the result of their protective effect against malaria (Ruwende et al. 1995; Sabeti et al. 2002; Tripathy and Reddy 2007; Louicharoen et al. 2009).

### Phylogenetic Analysis Reveals Introgression of Mitochondrial Genomes in East Population from Other Species

**Fig. 4.**
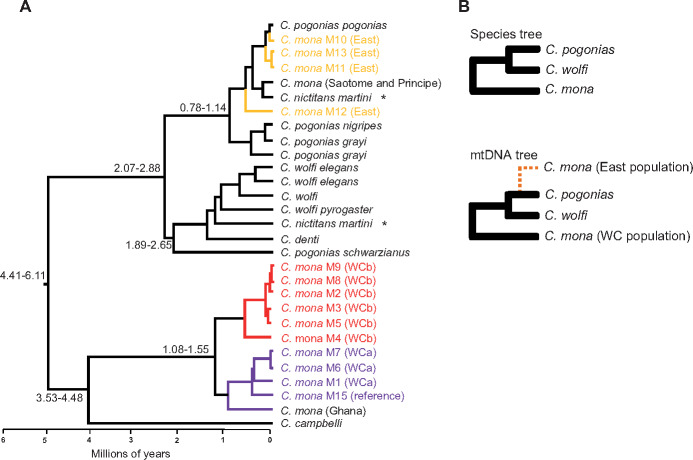
Phylogenetic relationships reveal hybridization of East population of *Cercopithecus mona* with others. (*A*) Phylogenetic tree of mitochondrial genomes. Details on phylogeny are provided in supplementary figure S10, Supplementary Material online. GenBank ID is provided in supplementary table S10, Supplementary Material online. Numbers beside nodes indicate 95% CIs of divergence time in unit of million years. Tip labels of *C. mona* consist of species codes followed by locality codes: East (Yellow) and West (WCa, Purple; WCb, Red). Individuals in black were downloaded from NCBI (supplementary table S10, Supplementary Material online). The two samples marked with asterisk are likely specimen mix-up as described in Guschanski et al. (2013). (*B*) Species tree according to previous studies based on mitochondrial DNA, X-chromosome sequences, Y-chromosome sequences, and karyotypes (Tosi et al. 2005; Moulin et al. 2008; Tosi 2017). Below is relationship according to mitochondrial phylogeny in (*A*).

Phylogenetic reconstructions based on the mtDNA genome using Bayesian inference and maximum-likelihood (ML) analyses obtained highly supported tree topologies (supplementary fig. S10 and table S11, Supplementary Material online), similar to topologies recovered elsewhere (Guschanski et al. 2013). The topology supported pervasive genetic introgression among African guenons, as previously observed (Guschanski et al. 2013). Particularly, within the *C. mona* species complex, *C. mona* individuals from the East population clustered together with *Cercopithecus pogonias* with very high bootstraps values and Bayesian posterior probabilities (PPs; node 12 in supplementary fig. S10 and table S11, Supplementary Material online). In contrast, all previous studies based on mitochondrial DNA, X-chromosome sequences, Y-chromosome sequences, and karyotypes have indicated a phylogenetic topology of (*C. mona*, (*Cercopithecus wolfi*, *C. pogonias*)) (fig. 4) (Tosi et al. 2005; Moulin et al. 2008; Tosi 2017). The parsimonious explanation for the unusual phylogenetic tree based on the *C. mona* mitochondrial genome is genetic introgression of the East population with other species (likely *C. pogonias*). This is unsurprising given *C. pogonias* shows geographical overlap with the East population of *C. mona* (fig. 2*A*). Divergence times between the East and WC populations inferred from the MCMCTREE and BEAST methods were 7.39 Ma (95% highest probability density: 4.00–14.44) and 5.65 Ma (95% highest probability density: 4.72–6.50), respectively (fig. 4 and supplementary figs. S10 and S11, Supplementary Material online), which were much older than that obtained from the nuclear genome (fig. 2*E*). Introgression may also have occurred in WCa or WCb given their older divergence time of mtDNA than nuclear genome. However, our evidence to support this scenario is limited and we cannot exclude other possibility, such as methodology differences, in estimating divergence time.

### Identification of Introgression Regions in East Population

**Fig. 5.**
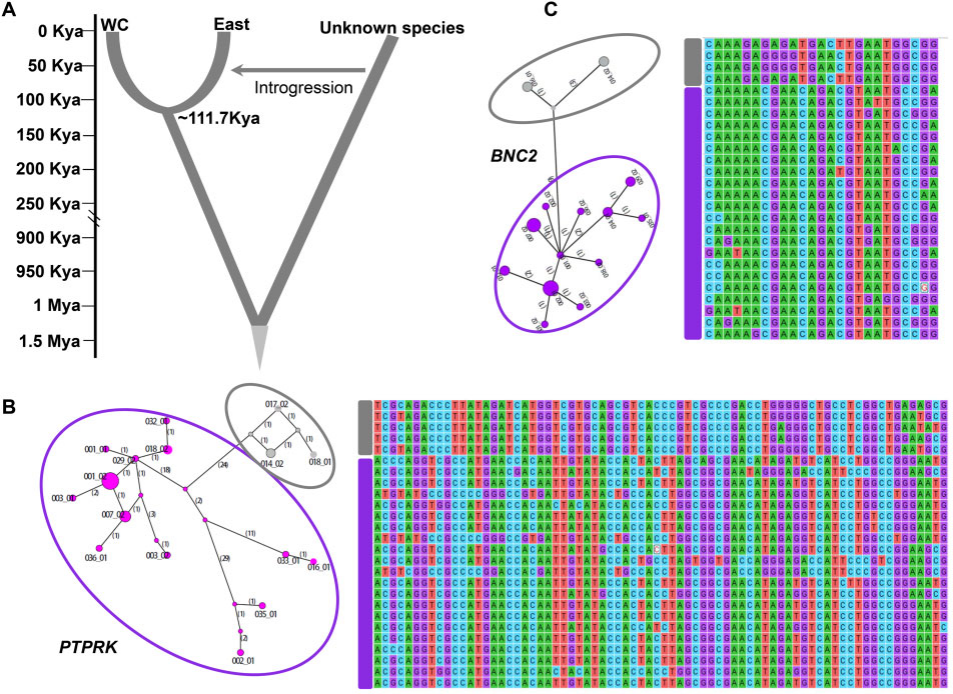
Analysis of introgressed regions. (*A*) Sketch showing introgression from an unknown *Cercopithecus mona* group lineage into East population of *C. mona*, with scale to the left indicating divergence time ka or Ma. (*B*, *C*) Genomic introgressed regions and median joining haplotype network tree of *PTPRK* and *BNC2*. Number in parentheses on each branch is mutation number. Haplotypes inside gray circle are potential introgressed haplotypes in East population, purple circle embodies other haplotypes. Right of each network is alignment of different haplotypes, with gray rectangle representing potential introgressed haplotypes in East population and purple rectangle representing other haplotypes.

Given the observed ancient hybridization of the mitochondrial genomes of the *C. mona* East population, we used the Hidden Markov Model (HMM) to deduce and categorize archaic introgression segments in the genomes of the East individuals (Skov et al. 2018). Using the constant mutation rate of 0.99e-09 per base pair (bp) per year derived from this study, this method inferred divergence between the East and WC populations occurring ∼111.7 ka (fig. 5*A*). However, this is a very rough estimate, as described in the original study (Skov et al. 2018), because the method only considers mutations, not gene flow or population size change (Skov et al. 2018). The archaic introgression length decreased when more strict PP cutoff values were applied. After applying a cutoff value of 0.98, the introgression length for different individuals ranged from 8.36 to 8.56 Mb (supplementary fig. S12 and table S12, Supplementary Material online). From these results, we identified an overlap region of ∼3.7 Mb, which may harbor candidate introgression genes shared among the East samples.

We randomly selected the same number of genomic regions (3,700 genomic regions with 1 kb in length) and then compared the nucleotide distance (dxy) between random and the putatively archaic introgression regions. Results showed that the latter exhibited a 2.08-fold higher nucleotide divergence than the former (0.0097 vs. 0.0046, supplementary fig. S13, Supplementary Material online). From these overlapping introgression regions, we identified 231 genes (supplementary table S13, Supplementary Material online). We only focused on the longest introgression segments because short archaic segments may be false positives (Skov et al. 2018). From the introgression regions longer than 10 kb, three genes were retrieved, that is, *VWA5B1* (von Willebrand factor a domain containing 5B1), *PTPRK* (protein tyrosine phosphatase receptor type k), and *FRAS1* (Fraser extracellular matrix complex subunit 1). The function of *VWA5B1* is not well documented in the literature, but the latter two genes are reportedly related to pigmentation in humans and other animals (Novellino et al. 2003; Sturm 2009). We constructed a haplotype network for these genes and observed a clear pattern in which the potential introgressed haplotype in the East population showed a large distance to other haplotypes (i.e., *PTPRK* in fig. 5*B*). In addition, among the 231 genes located at the introgressed regions, we also found two other genes, that is, *BNC2* (fig. 5*C*) and *EDN3*, which are related to pigmentation, showing signatures of genetic introgression in the East population (supplementary table S13, Supplementary Material online).

## Discussion

The establishment of high-quality reference genomes and population genomic data for primate species is necessary for study on primate evolution. The draft genome of *C. mona* presented here, with high-quality genome assembly and gene annotation, will serve as an ideal reference data point for future genome sequencing and comparative genomic studies. Our research is the first to evaluate the evolutionary history of *C. mona* from close geographical areas using mitochondrial and nuclear genomes based on next-generation sequencing.

### Incipient Speciation between WC and East Populations of *C. mona*

We took advantage of the high-depth whole-genome data set to infer the demographic history of *C. mona* using G-PhoCS, as well as changes in the effective population size using PSMC and MSMC2. Population genomics revealed that the WC and East populations diverged ∼84 ka, with no recent gene flow between them. The changes in present and ancestral effective population size corroborated long-term differentiation between the WC and East populations. These population genetic features rejected the hypothesis of panmixia between WC and East populations, similar to previous studies on incipient speciation. For example, Zhou et al. (2018) reported incipient speciation between two finless porpoise populations that diverged ∼40 ka without gene flow. Rivers are physical barriers associated with the distribution of certain taxa and allopatric diversification (Gascon et al. 2000). African rivers are considered important biogeographic barriers for various mammals (Nicolas et al. 2006, 2010), including within the *Crocidura olivieri* complex (Jacquet et al. 2015) and in other African mammals along the Volta and Congo-Ubangi rivers (Katuala et al. 2008; Nicolas et al. 2010). In Africa, several primate species have also been influenced by rivers, including *Mandrillus* sp., *Cercopithecus erythrotis*, *C. nictitans*, and *C. pogonias pogonias* (Grubb 2003; Clifford et al. 2004; Anthony et al. 2007; Harcourt and Wood 2012). The Ogooué River is proposed to have delimited the distribution of *Mandrillus sphinx* (Telfer et al. 2003), and the Sanaga River is an important factor influencing patterns of genetic diversity in chimpanzees from Cameroon (Mitchell et al. 2015). We propose that the major reason for this divergence could be because of the Niger and Benue rivers acting as a strong barrier to gene flow (fig. 2*A*), which have promoted incipient speciation in *C. mona*.

### Genetic Introgression in *C. mona* Species Complex

Due to the different patterns of inheritance, simultaneous analysis of nuclear and mitochondrial markers allows for a thorough evaluation of the evolutionary history of *C. mona*. Whole nuclear genomes estimated a divergence time of ∼84 ka between the East and WC populations. In contrast, the phylogenetic tree constructed by mitochondrial genome sequences revealed a higher differentiation between the East and WC populations, with a divergence time of ∼6.05 Ma. The East population clustered with *C*. *pogonias*, whereas the WC population clustered with other species (fig. 4). Unusual topology of the mtDNA sequences has been documented in other primate species (Tosi et al. 2004; Kuang et al. 2019), and in a recent study on Dryas monkeys (*Cercopithecus dryas*) (van der Valk et al. 2020). The parsimonious explanation is the probable occurrence of ancient hybridization. Prior studies have revealed frequent genetic introgression events between closely related primates (Detwiler et al. 2005; Tung and Barreiro 2017; Malukiewicz 2019), and confirmed the presence of admixture signatures in previously described primate hybrid zones (Cortes-Ortiz et al. 2007; Tung et al. 2008; Charpentier et al. 2012) and in new primate species complexes (Gligor et al. 2009). For example, de Manuel et al. (2016) identified introgression from bonobos to eastern and central chimpanzee subspecies, which are the closest geographically to the range of modern bonobos. Similarly, yellow baboons sampled with Anubis baboons near their range contain significantly higher levels of introgressed Anubis ancestry than those farther away (Charpentier et al. 2012; Wall et al. 2016).

In light of the species topology, the parsimonious explanation for the unusual phylogenetic tree based on mitochondrial genomes is the occurrence of genetic introgression in the East population with other species (fig. 4), although we cannot entirely exclude other scenarios. Thus, we further asked whether any functional genes or pathways have been introgressed in East population individuals from unknown species. Our evolutionary analyses identified several introgressed genes likely related to pigmentation, including *PTPRK* and *FRAS1*. *PTPRK* is a protein tyrosine phosphatase-κ gene showing expression in human primary keratinocytes, which are the source of peptides recognized by T cells in patients with melanoma (Novellino et al. 2003). The *FRAS1* gene encodes extracellular matrix proteins, which play critical roles in regulating membrane adhesion in the epidermal basement during embryonic development (Short et al. 2007). In addition to these two genes, another two genes (*BNC2* and *EDN3*) involved in pigmentation also exhibited signals of genetic introgression. *BNC2* encodes a protein that functions in skin color saturation and is associated with pigmentation in East Asian populations (Hider et al. 2013). *BNC2* expression and mutation levels are associated with facial pigment spots and have been widely studied in mice (Smyth et al. 2006; Vanhoutteghem et al. 2009) and zebrafish (Lang et al. 2009; Patterson and Parichy 2013). *EDN3* is involved in melanocyte development and pigmentation (Saldana-Caboverde and Kos 2010), and a variant of this gene can cause dermal hyperpigmentation in chickens (Dorshorst et al. 2011).

Haplotype networks of these pigmentation genes supported high divergence of introgression haplotypes in the East population with others from the WC population (fig. 5*B* and *C*). However, the functional consequences of the introgression of these pigmentation genes remain unclear. We propose that introgression of genes related to pigmentation may be related to color evolution in *C. mona*. Further ecological field surveys are necessary to detect any phenotypic differences between the East and WC populations, which will help in understanding the consequences of genetic introgression.

### Rapid Evolution of *G6PD* in African Guenons

In this study, we identified the *G6PD* gene as evolving under positive selection in the *C. mona* populations. *G6PD* provides instructions for the production of a glucose-6-phosphate dehydrogenase enzyme and its deficiency is related to malaria resistance. In modern humans, *G6PD*-deficiency mutations are widespread in specific geographic areas and are associated with resistance to malaria infection by *Plasmodium* parasites (Ruwende et al. 1995; Howes et al. 2012). Evolutionary studies have identified selection on the *G6PD*-deficient allele, believed to be the result of its malaria-protective effect in modern humans, like African and Southeast Asian (Ruwende et al. 1995; Sabeti et al. 2002; Tripathy and Reddy 2007; Louicharoen et al. 2009). Malaria is common in most African regions and one of the leading causes of death globally (Greenwood and Mutabingwa 2002; WHO 2015). It is also challenging for many animals in Africa. Until now, the biology of malaria infection in African guenons has been largely unstudied. For example, whether some populations are infected by malaria parasites or are resistant to malaria is unclear. We propose that rapid evolution of *G6PD* may be related to malaria resistance in some special African guenon populations; however, this requires further experimental validation.

### Rapid Evolution of SIV Infection-Related Genes in African Guenons

As a diverse group of lentiviruses, SIVs infect many primate species in sub-Saharan Africa (Pandrea et al. 2008; Ansari et al. 2014). Studies have suggested that primates have been infected with SIVs for millions of years (Keele et al. 2009; Rudicell et al. 2010; Etienne et al. 2011). Guenon species, such as *C. mona*, *C. nictitans*, and *C. cephus*, can be infected with SIVmon, SIVmus, and SIVgsn, respectively (Dazza et al. 2005; Schmokel et al. 2011). Studies suggest that these species-specific SIVs are the result of simultaneous host diversification, with concurrent splits occurring in SIV and primate lineages (host-dependent evolution) (Sharp et al. 2001). Evidence of this pattern has also been reported in African vervet monkeys (*Chlorocebus tantalus*, *Chlorocebus pygerythrus*, *Chlorocebus sabaeus*, and *Chlorocebus aethiops*) infected with species-specific SIVs. Using genome-wide population genomics, we identified two candidate SIV immunity genes, that is, *AKT3* and *IL13*, which showed evidence of positive natural selection in *C. mona*. The *Akt3* pathway plays a substantial role in the expression of Nef-mediated CCL5 (HIV-1 Nef is a protein expressed early in infection, which plays a major role in downregulation and viral pathogenesis) (Liu et al. 2014) and in different neurological mechanisms, such as proper growth and myelination during the course of infection (Easton et al. 2005; Tsiperson et al. 2013). *IL13* significantly downregulates CD8 expression in HIV-specific CD8(+) T cells. To enhance this downregulation, previous investigations proffered a novel IL-13R cytokine trap vaccine approach for HIV-1 (Ranasinghe et al. 2013). Interestingly, *IL13* has also been reported to play an important role in malaria resistance (Dewasurendra et al. 2012; Maiga et al. 2013). The rapid evolution of *AKT3* and *IL13* may be attributable to an “arms race” between SIV and monkey hosts. Within African guenons, *C. mona* may also be a potential biomedical animal model for future investigations on SIV infection, in addition to vervet monkeys.

## Materials and Methods

### Taxon Sampling

Animal collection approval and ethical clearance (NPH/GEN/121/XXV/561) for sample collection and transportation were obtained from the National Park Service of Nigeria. Import permission was approved by the National Forestry and Grassland Administration of China. Sampling followed the protocols of animal use authorized by the Kunming Institute of Zoology Animal Care and Ethics Committee. A total of 14 *C. mona* samples from different localities within three geographical regions in Nigeria as well as one *C. mitis* sample were collected for sequencing (fig. 2*A* and supplementary table S4, Supplementary Material online). Preliminary species identification was based on external morphology following previous study (Oates 2011). Samples were obtained via confiscation from poachers in the communities surrounding Nigerian national parks. Tissue samples collected from carcasses were preserved in 95% ethanol at −80 °C.

### Genome Sequencing and Assembly

One female *C. mona* sample was chosen for reference genome sequencing and assembly. Total genomic DNA was extracted from preserved muscle in ethanol using an QIAGEN extraction kit (Cat#13323, Blood & Cell Culture DNA Mini Kit). After assessing DNA quality, a library was prepared and sequenced on the Oxford Nanopore PromethION long-read sequencing platform. This generated a total of 156.3-Gb genomic data (∼54× coverage) with an average read length of 20.5 kb after removing the adaptor sequences and low-quality reads. Before genome assembly, NextDenovo (https://github.com/Nextomics/NextDenovo, last accessed June 2019) was applied to correct the high error rate of Nanopore long reads using the Illumina short reads. The corrected reads were then assembled using wtdbg v1.2.8 (https://github.com/fantasticair/wtdbg-1.2.8, last accessed June 2019), with the options wtdbg-1.2.8 -k 0 -p 23 -S 2, wtdbg-cns -c 3 -k 15, kbm-1.2.8 -k 0 -p 21 –S 2, and wtdbg-cns -k 13 -c 3. Preliminary genome assembly was further calibrated using NextPolish in NextDenovo. We used Benchmarking Universal Single-Copy Orthologs (Simao et al. 2015) to assess the completeness of our genome assembly and to search the assembly for annotated genes conserved among all mammals.

### Repeat Elements and Protein-Coding Gene Prediction

Repeat elements in the *C. mona* genome were annotated by both ab initio and homology-based approaches. We first developed a de novo repeat library using RepeatModeler v1.0.8 (Smit et al. 2008) with default parameters then used RepeatMasker v4.0.6 (Benson 1999) to identify known and de novo repeats by searching against the RepBase21.11 database (Bao et al. 2015) and de novo repeat library, respectively. RepeatProteinMask implemented in RepeatMasker was used to identify the TE-relevant proteins. In addition, we employed TRF v4.07 to predict the tandem repeats in the genome using default parameters. All identified repeat elements were classified into different categories (DNA, LINE, long terminal repeat, SINE, Unknown, and Other) according to the repeat database classification. Repeat annotations for the genome were combined into a nonredundant repeat annotation. In total, we predicted 41.32% of bases as TEs in the genome.

Protein-coding genes were also predicted by a combination of homology- and ab initio-based strategies. For homology-based prediction, the protein-coding sequences were obtained from the NCBI database for humans (*Homo sapiens*, GCA_000001405.28), chimpanzees (*Pan troglodytes*, GCA_002880755.3), gorillas (*Gorilla gorilla*, GCA_900006655.3), orangutans (*Pongo abelii*, GCA_002880775.3), and mice (*Mus musculus*, GCA_000001635.8). These protein sequences were then mapped to the *C. mona* genome using TBlastN v2.2.26 (Altschul et al. 1990) with an *E*-value cutoff of 1e-5. Proteins with multiple adjacent hits were connected to each other using genBlastA v1.0.4 (She et al. 2009). The aligned sequences and query proteins were then filtered and transferred to GeneWise v2.4.1 (Birney et al. 2004) to identify spliced alignments. Augustus v3.0.3 (Keller et al. 2011) was used for ab initio prediction, with optimized parameters trained from 1,000 randomly selected homologous genes. Finally, we integrated all gene sets to form a comprehensive and nonredundant gene set using in-house Perl scripts.

### Divergence Time Estimation

Gene sequences from six well-assembled primates (fig. 1) were downloaded from the NCBI database for comparative genomic analysis. For genes with numerous transcript isoforms, we only kept the longest for downstream analyses. Treefam (Ruan et al. 2008) was used to estimate gene families. We identified 8,574 one-to-one ortholog genes from these steps. Sequence alignments for each one-to-one ortholog were performed using MAFFT (Katoh et al. 2005). Phylogenetic relationships were constructed using RAxML v.8.1.15 (Stamatakis 2014) under the GTR + GAMMA model. Divergence times were inferred with MCMCTREE in PAML v4.8 (Yang 2007). To calibrate time, four calibrations were used: 1) *Homo sapiens*–*Macaca mulatta*: 23.0–33.9 Ma (Benton and Donoghue 2007), 2) Colobinae–Cercopithecinae: 8.5–23.03 Ma (Springer et al. 2012), 3) *Macaca*–*Papionini*: 5.5–23.03 Ma, and 4) a second calibration of *Chlorocebus*–*Cercopithecus*: 7.60–10.43 Ma (Pozzi et al. 2014). Calibrations were scaled to units of 100 My. The constraints for minimum and maximum bounds were soft, with a default 2.5% probability that bounds could be violated. Markov chain Monte Carlo (MCMC) was performed, with 10,000 generations as burn-in, then sampling every 100,000 generations until a total of 10,000 samples were generated. Two independent runs were executed, and convergence for each run to ensure an effective sample size (ESS) of more than 200 was determined in Tracer v1.6 (Rambault et al. 2014).

### Mutation Rate Estimation

The putative one-to-one orthologs between *C. mona* and humans identified above were used for mutation rate estimation. The yn00 program in the PAML 4.9e package (Yang 2007) was used to calculate synonymous distances (*Ks*) between orthologs. The formula “*r* = *Ks*/2*t*” (Wang et al. 2020) was applied to estimate the neutral substitution rate, where “*t*” is the mean divergence time between *C. mona* and humans. After the above steps, we obtained an average mutation rate of 0.99e-09 per bp per year, slightly smaller than the estimates reported by Pfeifer (2017) (0.99e-09 vs. 1.1e-09).

### Genome Resequencing and SNP Calling

From the samples (supplementary table S4, Supplementary Material online), 13 *C. mona* individuals and one *C. mitis* individual were used for whole-genome resequencing. Sequence libraries were designed in accordance with the Illumina library preparation framework and then sequenced on the Illumina Hiseq Xten platform to generate 150-bp paired-end reads using a 350-bp library. Raw sequence reads were mapped with BWA-MEM v0.7.12-r1039 (Li 2013) to the reference genome with default parameters. SAMtools v1.9 (Bigham et al. 2009) was used to sort and remove polymerase chain reaction duplicates. To reduce the number of miscalls around insertions/deletions (indels), the Genome Analysis Toolkit (GATK) v.2.6-5 (DePristo et al. 2011) was used to realign the raw gapped alignment. With the command “samtools mpileup -q 20 -Q 20 -C 50 -uDEf,” raw SNPs were called using SAMtools from locally realigned BAM files. To acquire high-quality SNPs for downstream analyses, the following filter criteria were used: 1) removal of sites around 6 bp from predicted indels, 2) removal of sites with consensus quality <40, 3) removal of sites with triallelic alleles and indels, and 4) retention of sites present in at least 95% of individuals. The filtered SNPs were phased using a read aware phasing approach implemented in SHAPEIT v2.0 (Delaneau et al. 2013). We used the following parameters to run SHAPEIT in read-conscious phasing mode: 200 conditional states, 10 iterations burn-in, 10 iterations pruning, 50 main iterations, and 0.5-Mb window size.

### Population Structure, Gene Flow, and Phylogeny

To exclude closely related samples from population structure analyses, we first estimated pairwise Identity-By-State scores among all samples by using PLINK v 1.90b5 (https://www.cog-genomics.org/plink/, last accessed October 2019). We found that M8 and M9 showed high pairwise genomic similarity (0.4988) thus leaving one of them (M8) in the downstream analyses. We used the Bayesian clustering method in Structure v2.3.4 (Pritchard et al. 2000) to infer the genetic ancestry of each sample by specifying the hypothetical ancestral clusters (*K*) from 1 to 5. We kept one SNP for each interval of 50 kb to avoid the linkage disequilibrium effect, resulting in 58,596 sites. Five independent runs were conducted, with the first 10,000 MCMC iterations treated as burn-in and the following 50,000 iterations then used. The most probable *K* value was estimated using Structure Harvester (http://taylor0.biology.ucla.edu/structureHarvester/, last accessed October 2019). PCA was analyzed using the GCTA v.1.24.2 package (Yang et al. 2011). To infer admixture events, we applied the *D*-statistic method in COMP-D (Mussmann et al. 2019). Based on a consensus-rooted four-taxon phylogeny, this method tests whether there is significantly different allele sharing between a source lineage (P3), and either of two receiving lineages (P1, P2) with reference to the outgroup (O). Here, to examine gene flow between the East and WC populations, we defined samples from the East population as P3, samples from WC as P1 and P2, and *C. mitis* as O.

Phylogenetic relationships were first estimated using the concatenated whole-genome SNPs with RAxML v.8.1.15 under the GTR + GAMMA model, with *C. mitis* as the outgroup (Stamatakis 2014). To minimize the effects of regions with strong natural selection and ancestral incomplete lineage sorting, we performed coalescent-based phylogenetic analyses using STAR (Liu et al. 2009) and ASTRAL v.4.10.8 (Mirarab et al. 2014) based on 10,000 putatively neutral 50-kb genomic windows by filtering out positions with repeat sequences, exons, and the 10-kb regions flanking them on each side. These windows should be at least 100 kb distant from each other. Individual gene trees for each window were constructed using RAxML v.8.1.15. Support values for each node were obtained using 100 rapid bootstrap replicates based on the GTR + GAMMA model.

### Demographic History Inference

The full-likelihood approach implemented in G-PhoCS v1.2.2 (Gronau et al. 2011) was used to infer the entire demographic history of *C. mona*, including ancestral population sizes, population divergence times, and migration rates. We randomly collected 1,000 loci (1,000-bp long) from the putatively neutral genomic region identified above for inference. We limited our data set to three individuals within each population due to computational constraints. To evaluate different migration scenarios among the different populations, as well as split time and goodness-of-fit model, six demographic models were established and compared (supplementary fig. S5 and tables S7 and S8, Supplementary Material online). The loci of the ghost population in Model 6 were set as “N.” The average log-likelihood value was obtained from MCMC and then used to calculate the AIC = −2 log-likelihood + 2P, where P is the number of parameters in the model. The model with the lowest AIC value was chosen as the best model. Every Markov chain was run for 3,000,000 iterations, with the first 1,000,000 iterations removed as burn-in, and thereafter sampling each 50th iteration. For the best model, we randomly choose an additional four independent data sets and reran G-PhoCS to check whether the estimated demographic parameters were stable. Convergence of each run was determined using Tracer v1.6.

Because G-PhoCS assumes a constant population size along phylogenetic branches, which may prevent the capture of gradual population size changes, for example, population bottlenecks, we adopted two other model-flexible methods: that is, PSMC (Li and Durbin 2011) and MSMC2 (Schiffels and Durbin 2014). PSMC and MSMC2 can be complementarily used as the former method exhibits better performance when inferring ancient histories (e.g., more than 20 ka), whereas the latter method is more reliable and well supported for recent population histories (Schiffels and Durbin 2014). The parameters for the PSMC analysis were -N25 -t15 -r5 -b -p “4 + 25 × 2 + 4 + 6.” A bootstrapping approach was carried out with 100 replicates to evaluate variation in the inferred effective population size (Ne) trajectories. For MSMC2 analysis, we selected three individuals with the highest sequencing coverage from each group of interest. As MSMC2 requires haplotypes as input, the phased VCF file from SHAPEIT was used. Other input files were prepared using the scripts provided in GitHub (https://github.com/stschiff/msmc-tools, last accessed October 2019) and MSMC2 was run using default parameters. We used a generation time of 8.5 years (Anandam et al. 2013) and a neutral mutation frequency of 0.99e-09 per site per year (as estimated above). A 50% elative cross coalescent rate was used as a rough estimate of divergence time (Malaspinas et al. 2016; Wang et al. 2020).

### Mitochondrial Genome Assembling and Alignment

Mitochondrial genome sequence of each individual was obtained from Illumina Hiseq Xten reads by using NOVOplasty 2.4 (Dierckxsens et al. 2017). *K*-mer was set to 33 and one mitogenome of *C. mona* (JQ256979) download from GenBank was used as bait reference. Assembled contigs were annotated with MITOS (http://mitos2.bioinf.uni-leipzig.de/index.py, last accessed October 2019). The reliability of mitochondrial contig assemblies was further validated via BLAST searches against the reference mitgenome (JQ256979). Sequences were aligned using MUSCLE v3.8.31 (Edgar 2004), as implemented in MEGA v7.0.14 (Kumar et al. 2016). Protein-coding genes were translated to amino acid sequences to ensure an open reading frame and avoid amplification of NUMTs (nuclear mitochondrial DNA segments), as NUMTs have many stop codons in the sequences.

### Mitochondrial Phylogeny and Divergence Time

Including the outgroup *C. mitis*, we generated 15 near complete mitochondrial genome sequences in this study. For phylogenetic reconstructions, additional sequences were added from GenBank (supplementary table S10, Supplementary Material online) to expand our data sets covering mtDNA genomes from a total of 79 African guenon samples. We did not include the d-loop sequences due to their low quality. We generated alignments for individual loci with MEGA 7.0 (Kumar et al. 2016). Indels and badly aligned positions were removed using standard settings with Gblocks v0.91b (Castresana 2000). The aligned genomes were partitioned into protein-coding genes, noncoding fragments, rRNAs, and tRNAs. The protein-coding genes were further partitioned into first, second, and third codon positions using Split Codons (Stothard 2000). We used PartitionFinder v2.1.6 to evaluate the best partitioning scheme and to determine replacement models for each partition using corrected AIC (AICc) (Lanfear et al. 2012). ML analysis was conducted in RAxML v.8.1.15 with 1,000 bootstrap replications under the best partition scheme (https://embnet.vital-it.ch/raxml-bb/) (Stamatakis 2014). Bayesian inference analysis was performed using MrBayes v3.1.2 (Ronquist and Huelsenbeck 2003). We conducted two independent runs using four MCMC runs, each for 10,000,000 generations, with tree sampling every 1,000 generations. Tracer v1.6 (Rambault et al. 2014) was used to assess convergence visually and to verify that the ESS was >200. The posterior distribution of trees was summarized after checking for congruence by removing the first 25% of generations as burn-in. Lineages with Bayesian PP and posterior branch support values higher than 0.95 were considered strongly supported (Erixon et al. 2003).

Divergence time was estimated by MCMCTREE and BEAST v1.8.0 (Drummond and Rambaut 2007), respectively. In the MCMCTREE analysis, we included our data as 17 partitions according the PartitionFinder results. Parameters and calibrations were the same as used in comparative analysis. For BEAST analysis, a lognormal relaxed clock was used to estimate branch lengths, and the Yule model was used for the tree as priors. We performed four independent MCMC runs, each with 20,000,000 million generations, and with trees and parameters sampled every 2,000 generations. The burn-in and convergence of chains were assessed using Tracer v1.6 (Rambault et al. 2014), with a burn-in of 25%, to verify that the ESS was >200. We used LogCombiner v1.8.3 to combine results of the log file independent runs and Tree Annotator 2.4.4 was implemented to create a standard tree with maximum clade credibility using median node heights in BEAST. The tree was visualized, summarized, and edited using FigTree v1.4.2 (Rambaut 2012).

### Analysis of Introgression

We adopted a newly developed HMM method (Skov et al. 2018) to infer the signature of archaic introgression in the East *C. mona* group. This approach can identify a high density of foreign nucleotide variants without a reference genome and shows a lower false positive rate than that of other methods, such as S* (Vernot and Akey 2014; Vernot et al. 2016) and Sprime (Browning et al. 2018). The WCa + WCb individuals were treated as an outgroup. The callable regions were identified using the repeat masked genome and then classified into bins of 1,000 bp. The number of variants found in the outgroup was calculated using VCFtools v0.1.11 (Danecek et al. 2011). In 100-kb windows, the background mutation rate was estimated using the variant density of all variants in the outgroup population. HMM was configured with a set of starting parameters based on human parameters (Skov et al. 2018). To ensure accuracy of the final parameter data, we designed the model across five separate runs, varying the starting parameters. The posterior decoding then specified whether consecutive 1-kb windows changed or maintained their condition (“archaic” or “nonarchaic”), dependent on PP.

### Inference of High-Divergence Regions and Selection Signals between East and WC Populations

Under topological transformation outlined previously (Wang et al. 2018), genomic differentiation was determined based on PBS (Yi et al. 2010). The PBS value calculates the extent of sequence shift in a population branch as the divergence between it and other branches of a population tree. First, using Weir and Cockerham’s method in VCFtools v0.1.11 (Danecek et al. 2011), *F*_ST_ pairwise statistics were calculated with nonoverlapping 50-kb genomic windows. Negative *F*_ST_ values were treated as 0. Log-transformed *F*_ST_ values were used for PBS calculation (Yi et al. 2010; Wang et al. 2018). The upper 1% of each PBS distribution was described as the high divergence regions (HDRs). Typically, regions that have experienced selection will show significantly decreased levels of nucleotide diversity and long-range homozygosity of the haplotype (Sabeti et al. 2006). To check for signatures of selective sweeps in HDRs, we first calculate the nucleotide diversity (Pi) of each window using VCFtools v 0.1.11. The XP-EHH values (haplotype-based statistics) were estimated using selscan v1.1.0a (Szpiech and Hernandez 2014) assuming 1 Mb = 1 cm across the *C. mona* genome. By subtracting the genome-wide mean XP-EHH and dividing by the standard deviation, the XP-EHH values were normalized. The average XP-EHH values of SNPs in each window were calculated by an in-house Perl script. Gene Ontology enrichment was analyzed with DAVID (Database for Annotation Visualization and Integrated Discovery) (Dennis et al. 2003). Only Gene Ontology terms with a *P* value of <0.05 were considered.

## Supplementary Material

Supplementary data are available at *Molecular Biology and Evolution* online.

## Supplementary Material

msaa248_Supplementary_Data
