# Supplementary material for: Population Genomics Reveals Incipient Speciation, Introgression, and Adaptation in the African Mona Monkey (Cercopithecus mona)
Source: Mol Biol Evol. 2020 Sep 28;38(3):876–90. doi: 10.1093/molbev/msaa248 (PMC7947840; doi:10.1093/molbev/msaa248)
Supplement: msaa248_Supplementary_Data [file msaa248_supplementary_data.pdf]

SUPPLEMENTARY FILE

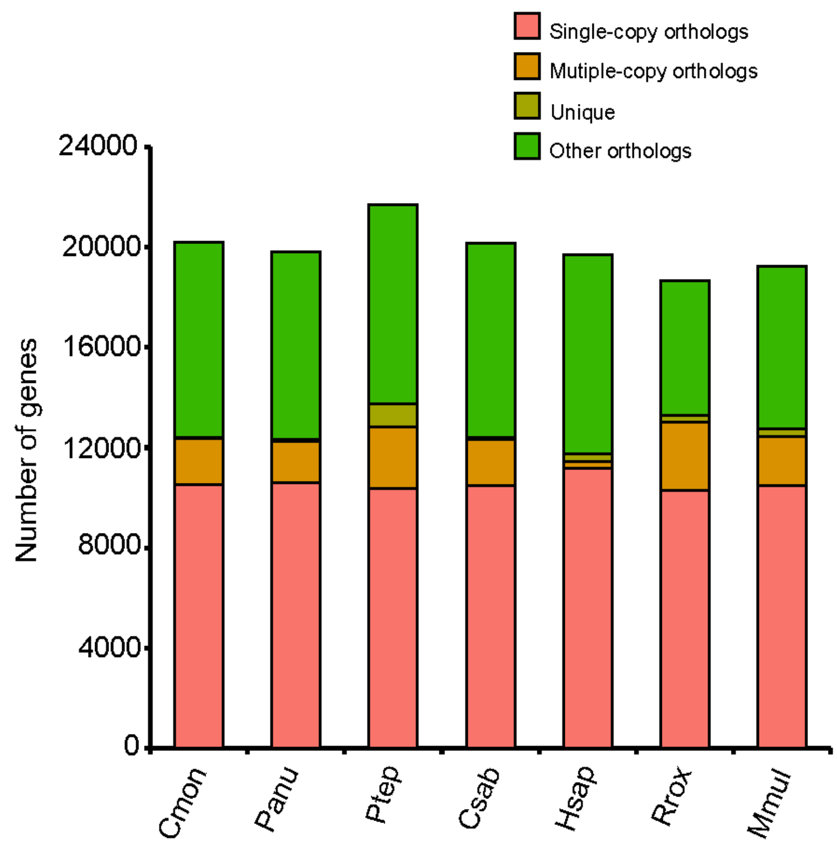

Supplementary Figure S1: Barchart distribution of single-copy, multiple-copy, unique and other orthologs genes in *C. mona* and six closely related primates. The number of single-copy, multiple-copy, unique and other orthologs genes found in each species is shown, along with the number of genes indicated on the y-axis. "Single-copy orthologs" means genes are present in all species but have only one copy in the target species. "Mutiple-copy orthologs" means genes are present in all species but the target species have more than 1 copies. "Unique" means gene only present in target species. "Other orthologs" are genes that are present in at least two species, but not in all species. *Cercopithecus mona* (Cmon), *Papio anubis* (Panu), *Chlorocebus sabeaus* (Csab), *Homo sapiens* (Hsap), *Piliocolobus tephrosceles* (Ptep), *Rhinopithecus roxellana* (Rrox), *Maccaca mulata* (Mmul).

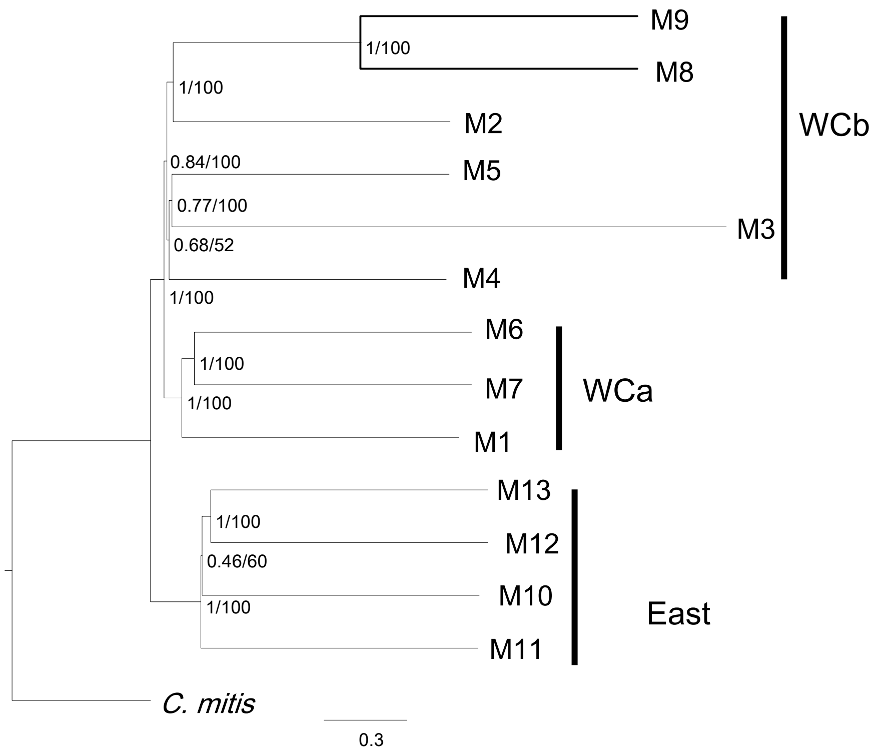

Supplementary Figure S2: Coalescent-based phylogeny of *C. mona* individuals (M1-M13) infered by ASTRAL based on 10,000 putatively neutral 50 kb genomic windows. Branch lengths are in coalescent units as estimated by ASTRAL. Numbers at nodes refer to bootstrap values (ASTRAL) and posterior probabilities (STAR), respectivly.

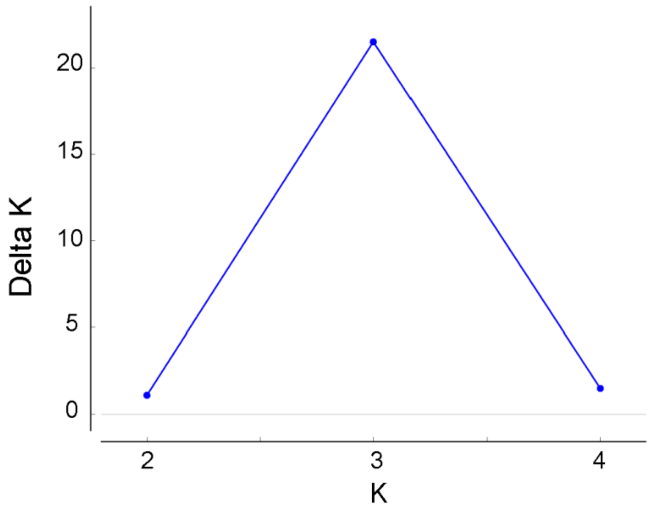

Supplementary Figure S3: The values of delta K in the STRUCTURE analysis show maximal value at K = 3 genetic groups.

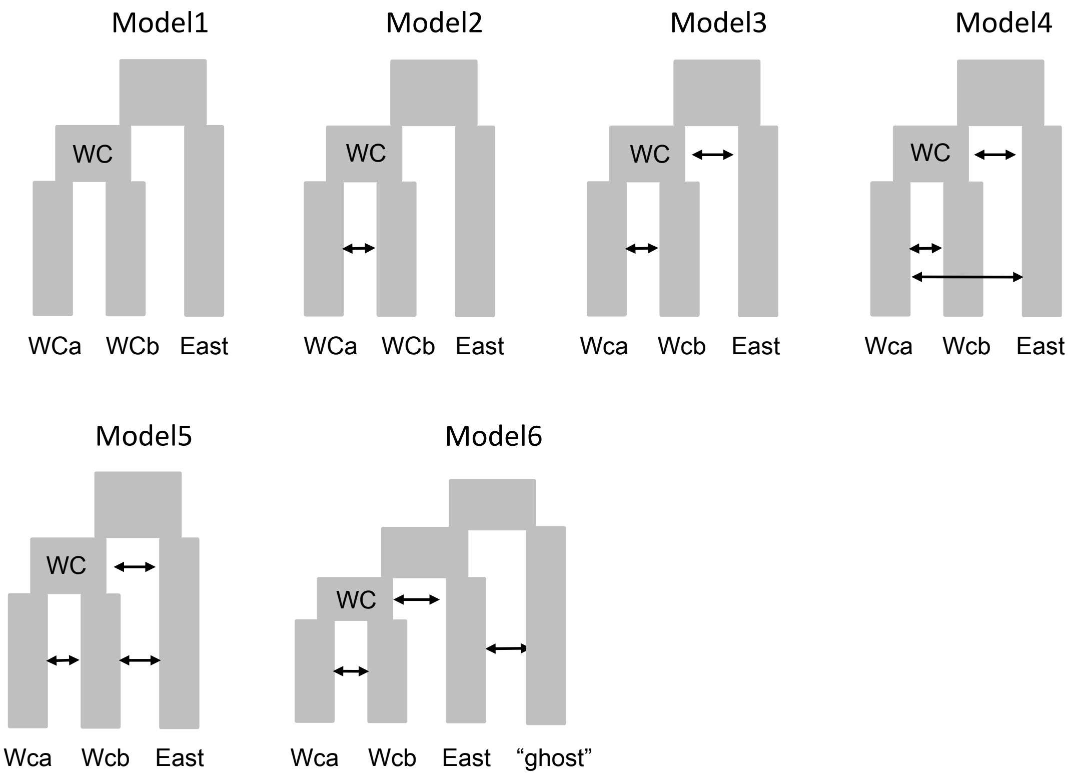

Supplementary Figure S4: Models of different migration scenario between populations and split time by G-PhoCS program.

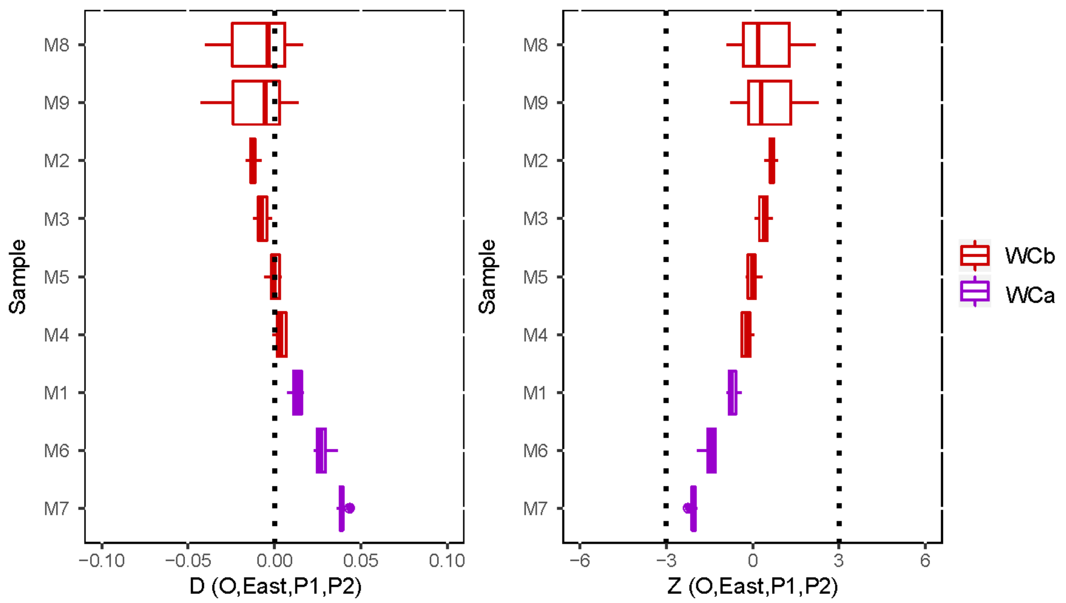

Supplementary Figure S5: Box plot representing D-statistics (left) and Z score (right) for each group (Outgroup, East, P1, P2). We test whether there is statistically significant different allele sharing between a source lineage (East), and either of two receiving lineages (WCa and WCb) with reference to the outgroup (O). P1 is each individual at Y- axis, and P2 denotes the individuals from the other receiving lineages. Z score with absolute value bigger than 3 was considered as significant.

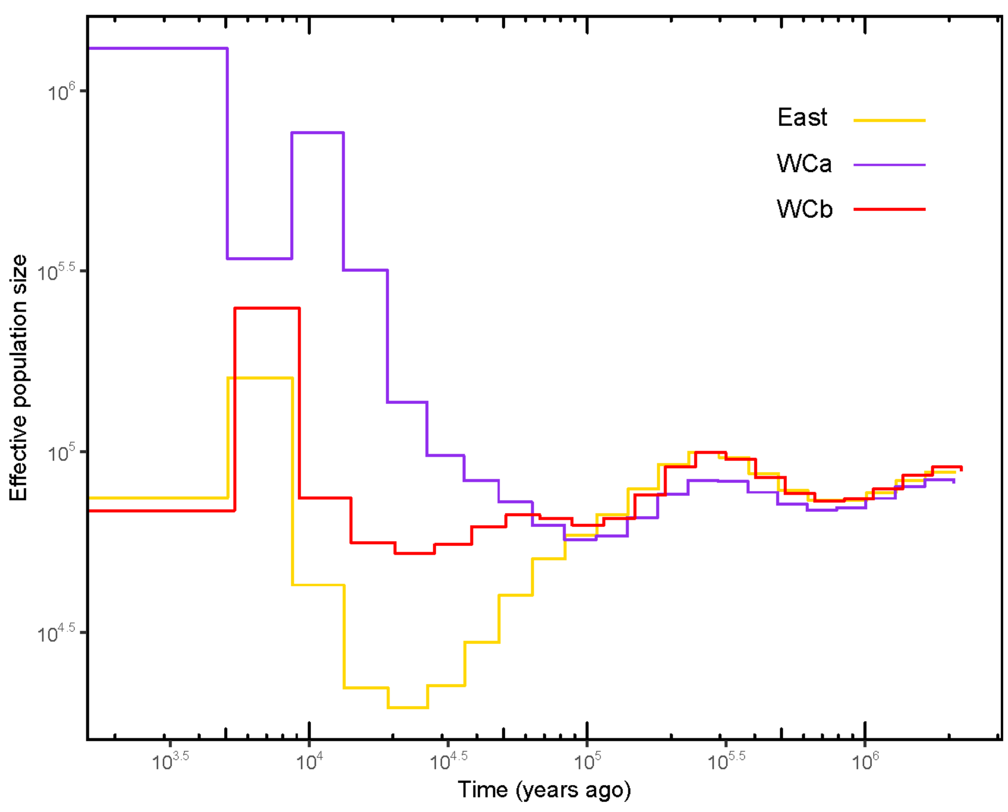

Supplementary Figure S6: Effective population size (Ne) change inferred using MSMC2. Plots are scaled using a generation time of 8.5 years and an autosomal mutation rate of 8.415×10^{-9} per base pair per generation.

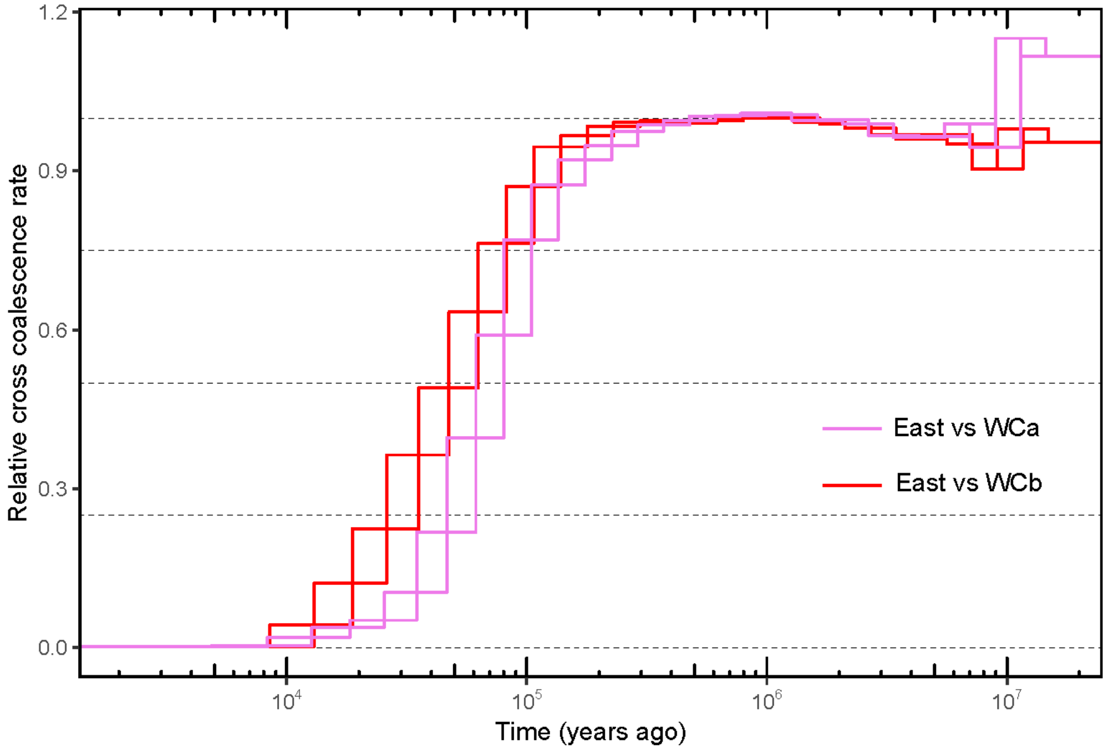

Supplementary Figure S7: Divergence time between East population and WC population was estimated by MSMC2. RCCR value close to "1" indicates two populations are well mixed while "0" indicate fully separated. The 50% RCCR can be viewed as a rough estimate of the divergence time.

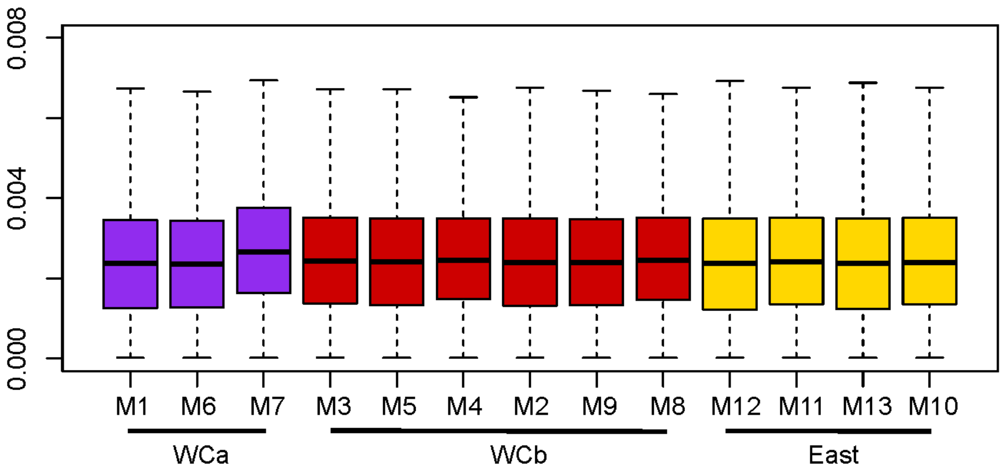

Supplementary Figure S8: Genetic diversity for different individuals. Y-axis represents the ratio of average nucleotide differences between a pair of chromosomes in 50Kb windows. Colors match the three groups as indicated in Figure 2B.

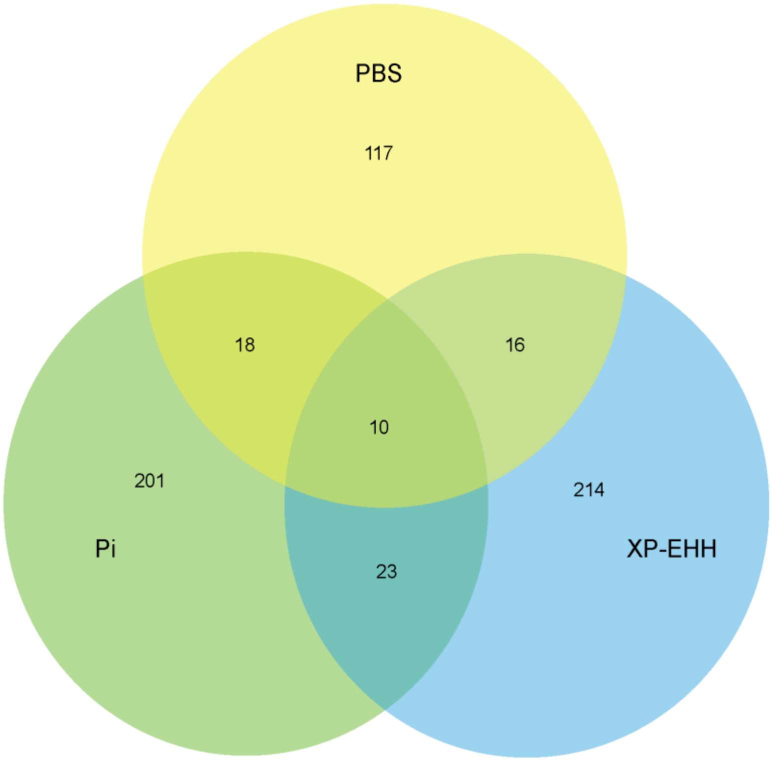

Supplementary Figure S9: Venn diagram showing the numbers of candidate positively selected genes inferred by different statistics: PBS, Pi and XP-EHH. The extreme high value (top 1%) in PBS and XP-EHH statistics and the extreme low value (low 1%) in Pi statistic were used as the cut-off value to identify the candidate selected gene.

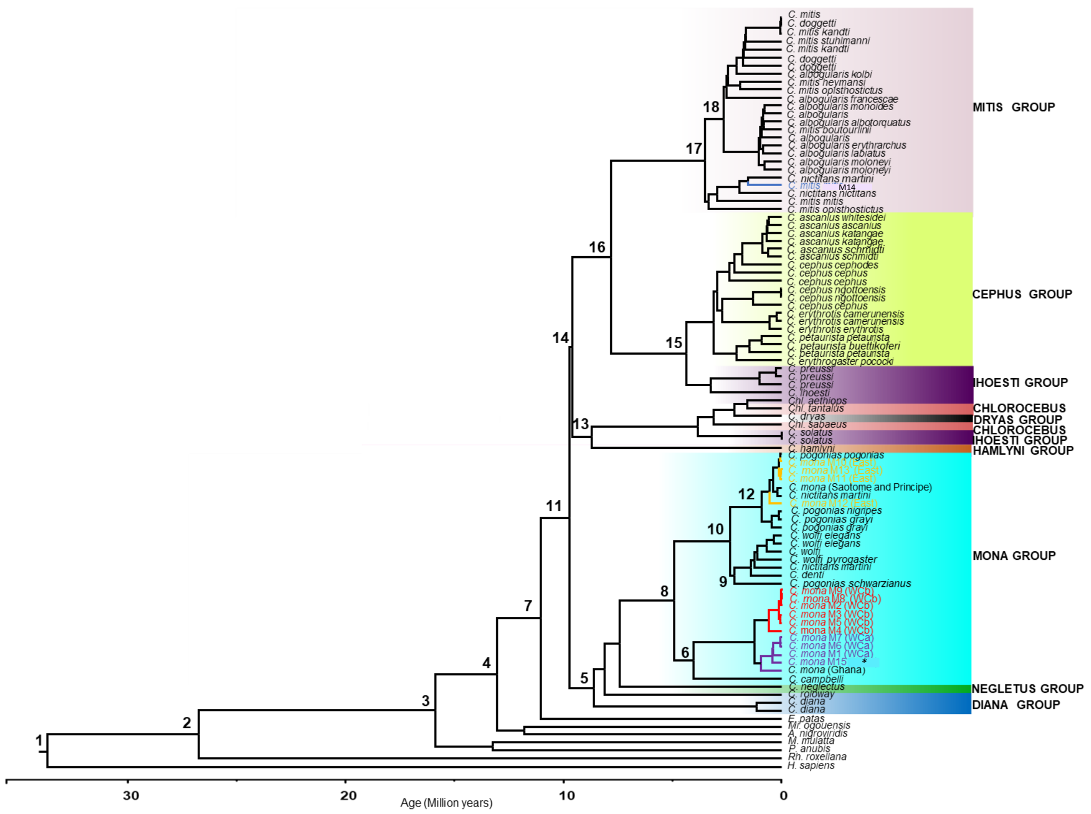

Supplementary Figure S10: Phylogenetic relationship inferred from mitochondrial genomes. The detail information at the nodes (with number 1- 18) including Maximum-likelihood bootstraps values (BP), Bayesian posterior probabilities (PP), mean divergence and 95% composite credibility intervals (CI) in million years (myr) are presented in supplementary Table S11. Tip labels of *C. mona* consists of species codes followed by the locality codes, East (Yellow), West central (WCa, Purple; WCb, Red), Nigeria. Colour bars on the species names refer to the species groups. Individuals in Black were downloaded from NCBI (Supplementary Table S10).

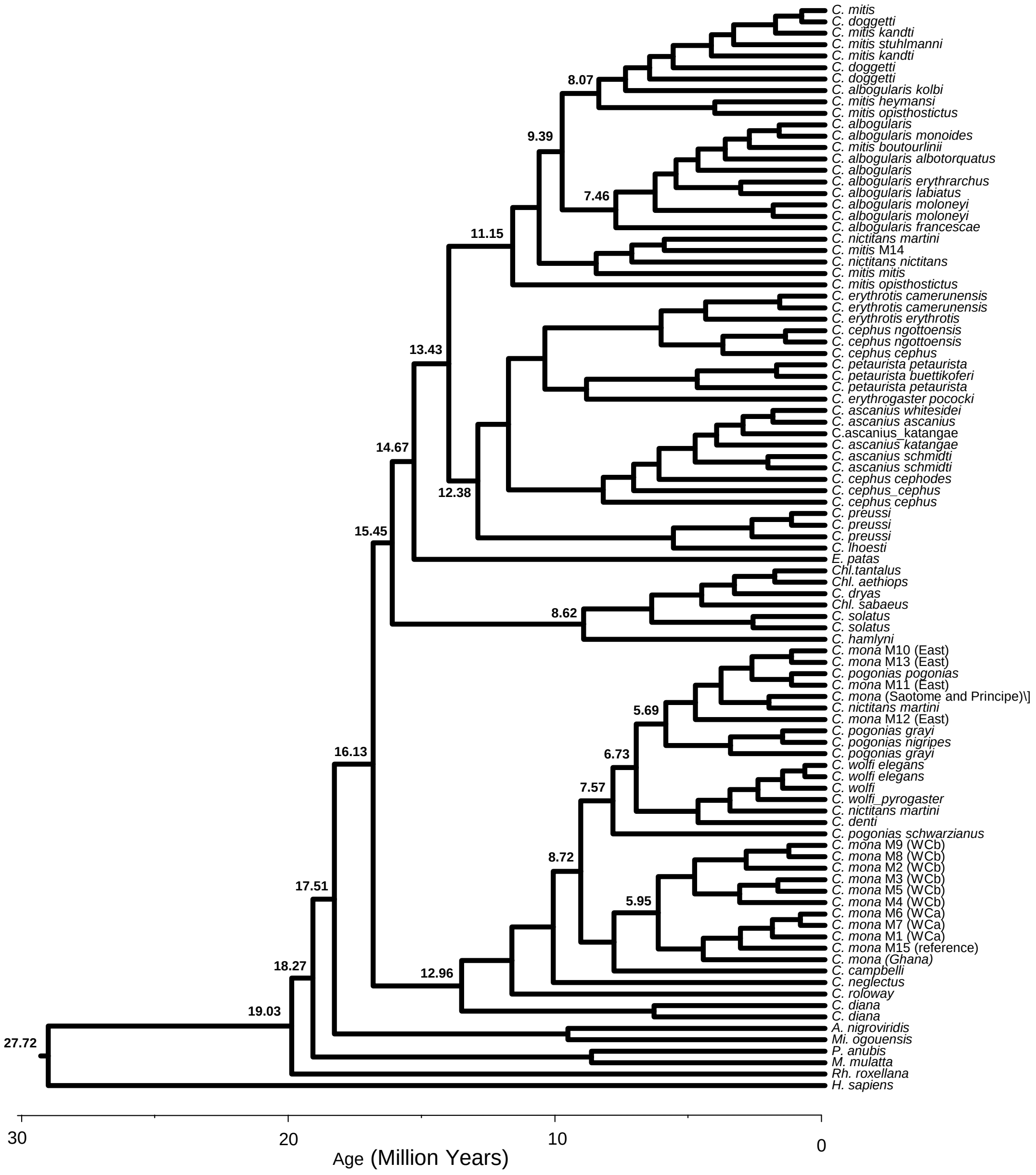

Supplementary Figure S11: Mitochondrial divergence time inferred by MCMCTREE program. Numbers besides the node indicate the divergence time of interest.

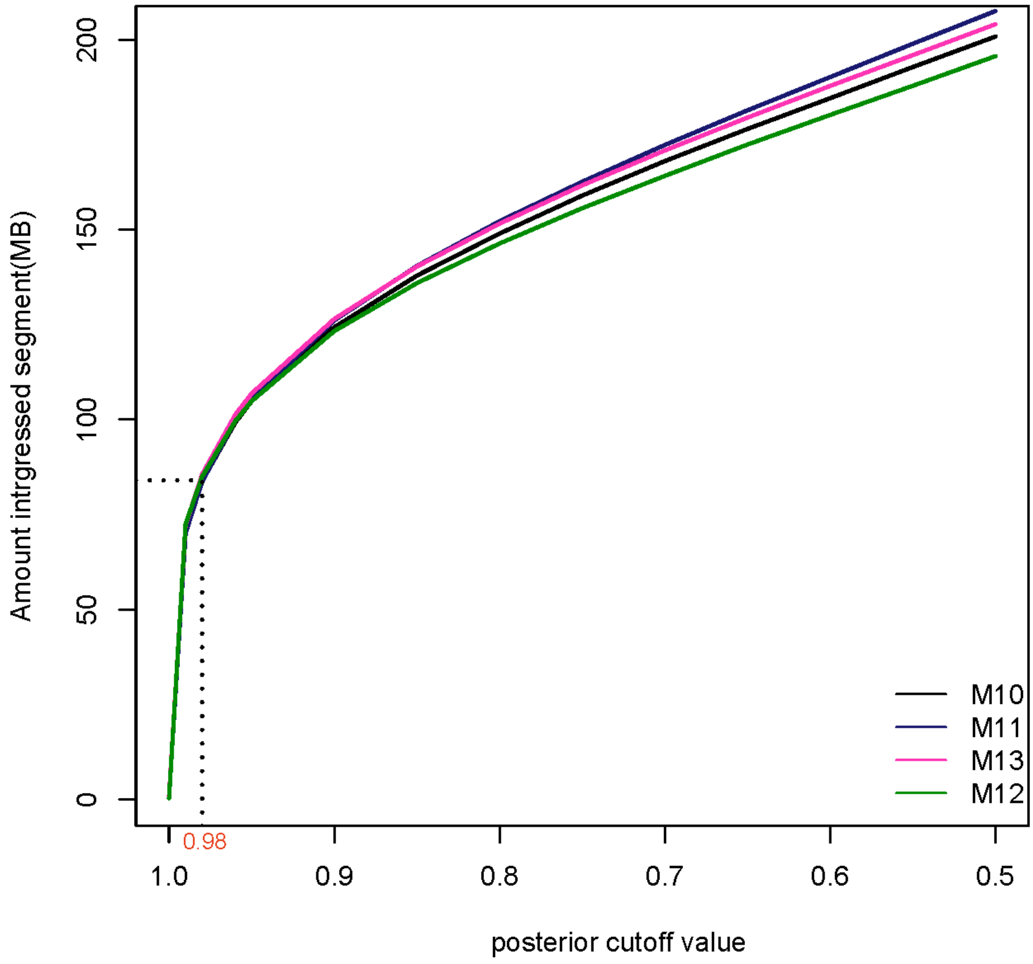

Supplementary Figure S12: Introgressed length from other Mona group lineage into individuals (M10-M13) of East clade when different posterior cutoff values were applied. The dotted line represents the number of segments (MB) with the posterior probability cutoff (0.98 highlighted) shown on the x-axis.

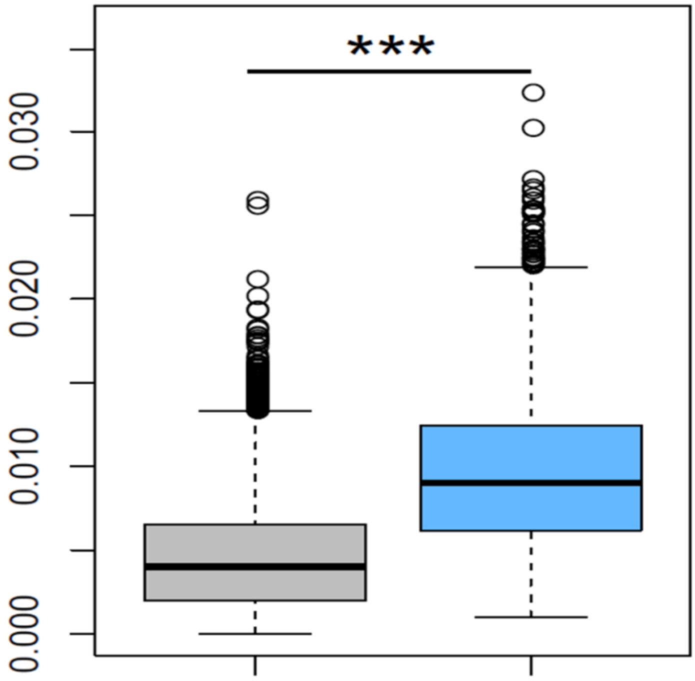

Supplementary Figure S13: Comparison of the nucleotide distance (dxy, Y- axis) between introgressed (light blue) and non-introgressed (gray) regions between East and West clades. Asterisks denote the level of significance by two-tailed Mann-Whitney test (*** p <2.2e-16).

Supplementary Table S1: Assembly statistics of the *C. mona* reference genome

Stat Type	Contig Length (bp)	Contig Number
N50	22,791,723	36
N60	18,349,773	50
N70	14,205,761	68
N80	9,558,045	93
N90	4,142,170	137
Longest	81,511,750	1
Total	2,902,804,697	1889
Length>=5kb	2,902,804,697	1889

Supplementary Table S2: Genome assessment based on BUSCO annotations

**Type**	**Number**	**Percent (%)**
Complete BUSCOs (C)	3,827	93.2
Complete and single-copy BUSCOs (S)	3,769	91.8
Complete and duplicated BUSCOs (D)	58	1.4
Fragmented BUSCOs (F)	107	2.6
Missing BUSCOs (M)	170	4.2
Total BUSCO groups searched	4104	-

Supplementary Table S3: Repetitive element annotation in the genome assembly

Type	Length (bp)	% in genome
DNA	76,352,010	2.630284
LINE	536,610,509	18.48593
SINE	386,222,483	13.30515
LTR	218,483,782	7.526644
Other	136	0.000005
Unknown	63,380,885	2.183436
Total	1,199,388,864	41.31828

Supplementary Table S4: Details about samples used for genome sequencing

ID	KIZ ID	Specie Name	Number of Reads	Depth	Mappable Coverage	Sex	Tissue	Location	Latitude	Longitude	Collection Date
M3	YP180528-029	*C. mona*	425,617,143	23	98.63%	Female	Muscle	Central Nigeria Okomu (Tree house)	N6.2151	E 5.24000	12/2017
M4	YP180528-007	*C. mona*	429,963,950	23	99.15%	Female	Muscle	Central Nigeria Okomu (Tree house)	N6.2280	E5.31113	02/2018
M9	YP180528-032	*C. mona*	459,688,692	25	98.37%	Female	Muscle	Central Nigeria Okomu (Udo)	N6.3121	E5.36124	03/2018
M2	YP180528-036	*C. mona*	449,120,176	24	98.66%	Male	Muscle	Central Nigeria Okomu (Udo)	N6.2340	E 5.38131	03/2018
M5	YP180528-035	*C. mona*	440,719,118	24	98.70%	Female	Muscle	Central Nigeria Okomu (Udo)	N6.2391	E5.39511	03/2018
M8	YP180528-033	*C. mona*	406,370,615	22	98.60%	Female	Muscle	Central Nigeria Okomu (Tree house)	N6.2240	E5.29000	04/2018
M13	YP180528-018	*C. mona*	432,158,692	23	98.63%	Female	Muscle	East Nigeria Crossriver (Akampa)	N5.21901	E8.26300	04/2018
M6	YP180528-002	*C. mona*	404,546,976	22	97.05%	Female	Muscle	West Nigeria Ogun (Ibese)	N6.9134	E2.91332	02/2018
M7	YP180528-003	*C. mona*	444,366,582	24	97.22%	Male	Muscle	West Nigeria Ogun (Ibese)	N6.8943	E2.90339	02/2018
M11	YP180528-016	*C. mona*	572,933,971	31	98.62%	Female	Muscle	East Nigeria Cross River (Akampa)	N5.21816	E8.26280	02/2018
M10	YP180528-017	*C. mona*	526,735,084	28	98.58%	Male	Muscle	East Nigeria Crossriver (Akampa)	N5.21817	E8.26283	02/2018

M12	YP180528-014	*C. mona*	470,203,112	25	98.43%	Male	Muscle	East Nigeria Cross River (Akampa)	N5.21814	E8.26278	02/2018
M1	YP180528-001	*C. mona*	478,009,395	26	93.63%	Female	Muscle	West Nigeria Ogun (Ibese)	N6.8939	E2.90334	03/2018
M14*	YP180528-023	*C. mitis*	457,859,907	25	98.94%	Female	Muscle	East Nigeria Cross River (Akampa)	N5.22466	E8.26290	01/2018
M15*	YP180528-039	*C. mona (reference)*	The individual used for genome assembly.			Female	Muscle	East Nigeria Cross River (Akampa)	N5.25752	E8.31500	03/2019

*Represents individuals with confiscated locality name, original locality is unknown.

Supplementary Table S5: The results of likelihood-ratio tests and AIC comparisons for six demographic models in supplementary Figure S4

**Models**	**P**	**logL**	**AIC**
Model1	7	-1399.72	2813.44
Model2	9	-1396.42	2810.84
Model3	11	-1355.15	2732.30
Model4	13	-1375.43	2776.86
Model5	13	-1375.09	2776.19
Model6	15	-1374.82	2779.64

Note that Model6 was hard to achieve convergence despite we have elevated the MCMC runs to 5,000,000 iterations.

Supplementary Table S6: Demographic parameters inferred by Generalized Phylogenetic Coalescent Sampler (G-PhoCS) software

Parameter	Mean	Lower 95% CI	Upper 95% CI
θ_{East}	37551.99	31684.49	43481.88
θ_{WCa}	15983.36	3098.63	32566.84
θ_{WCb}	22459.89	4049.32	45935.83
θ_{WC}	150207.96	117032.09	187685.68
$\theta_{WC\text{-}East}$	98068.92	94432.56	101622.10
$T_{WCa\text{-}WCb}$	6696.97	1060.61	13979.80
$T_{WC\text{-}East}$	84404.04	63151.52	104343.43
$M_{WCa\text{->}WCb}$	1.48%	0.17%	3.90%
$M_{WCb\text{->}Wca}$	1.66%	0.19%	4.37%
$M_{WC\text{->}East}$	20.12%	11.05%	31.33%
$M_{East\text{->}WC}$	20.29%	11.15%	31.61%

Supplementary Table S7: G-PhoCS results from other four independent results.

Parapeters	Rep1 Mean(95%CI Lower-95%CI Upper)	Rep2 Mean(95%CI Lower-95%CI Upper)	Rep3 Mean(95%CI Lower-95%CI Upper)	Rep4 Mean(95%CI Lower-95%CI Upper)
$\boldsymbol{\theta}_{East}$	40891.27(32147.95-49976.23)	39869.28(32094.47-47002.38)	41434.94(35567.44-47614.38)	36633.99(30816.99-42857.99)
$\boldsymbol{\theta}_{WCa}$	16096.26(3755.79-39076.65)	21004.16(3633.39-43374.93)	14284.02(3055.91-39554.37)	17254.90(5184.19-39916.82)
$\boldsymbol{\theta}_{WCb}$	20187.17(3618.54-41574.57)	20918.00(3137.25-43383.84)	24732.62(3975.04-51016.04)	26776.59(7712.42-48395.72)
$\boldsymbol{\theta}_{WC}$	132284.61(102079.62-165965.54)	133333.33(102394.53-164946.52)	139456.33(110956.63-169322.64)	125724.90(99236.48-159489.01)
$\boldsymbol{\theta}_{WC\text{-}East}$	91595.37(88217.47-95190.14)	96417.11(92881.76-99964.35)	92439.10(89058.23-95959.60)	96232.92(93205.59-99806.89)
$\mathbf{T}_{WCa\text{-}WCb}$	7630.20(1353.54-15252.53)	6246.16(818.18-12979.80)	6803.84(1048.48-12040.40)	7680.61(2090.91-12868.69)
$\mathbf{T}_{WC\text{-}East}$	88617.17(63898.99-112828.28)	72790.91(54030.30-91757.58)	94426.26(74404.04-114040.40)	78024.24(63030.30-93010.10)
$\mathbf{M}_{WCa\text{->}WCb}$	1.47%(0.15%-3.24%)	1.10%(0.08%-3.23%)	1.24%(0.14%-3.19%)	1.77%(0.30%-4.14%)
$\mathbf{M}_{WCb\text{->}Wca}$	1.46%(0.04%-6.12%)	1.32%(0.09%-3.88%)	1.41%(0.16%-3.62%)	1.64%(0.28%-3.82%)
$\mathbf{M}_{WC\text{->}East}$	18.42%(10.13%-26.20%)	13.07%(5.10%-23.32%)	19.54%(11.66%-29.18%)	14.85%(7.29%-24.48%)
$\mathbf{M}_{East\text{->}WC}$	23.71%(12.53%-37.86%)	20.24%(10.95%-31.19%)	22.79%(12.59%-34.03%)	18.73%(7.73%-28.05%)

Supplementary Table S8: Mean value of Z-score derived from individual tests by D-statistics and p-value. *Cercopithecus mitis* was used as outgroup, samples from East treated as P3 and samples from WC treated as P1 and P2

**Model**	**Z-score**	**p-value**
(Outgroup, East, WCa, WCb)	1.07213	0.283661

Supplementary Table S9: Gene Ontology analysis of genes located in regions that strongly differentiated East from WC

Category	Term	No. of genes	*p* value
Cluster 1	Kinase	17	0.0001
	Nucleotide-binding	27	0.0006
	binding site:ATP	13	0.0010
	Transferase	25	0.0015
	IPR008271:Serine/threonine-protein kinase, active site	9	0.0028
	GO:0005524~ATP binding	23	0.0028
	GO:0016301~kinase activity	8	0.0029
	ATP-binding	21	0.0030
	nucleotide phosphate-binding region:ATP	17	0.0030
	domain:Protein kinase	11	0.0031
	IPR000719:Protein kinase, catalytic domain	11	0.0043
	IPR011009:Protein kinase-like domain	11	0.0078
	Serine/threonine-protein kinase	9	0.0086
	IPR017441:Protein kinase, ATP binding site	9	0.0091
	SM00220:S_TKc	9	0.0131
	GO:0004674~protein serine/threonine kinase activity	8	0.0284
	active site:Proton acceptor	11	0.0286
Cluster 2	h_tollPathway:Toll-Like Receptor Pathway	5	0.0006
	hsa04620:Toll-like receptor signaling pathway	6	0.0041
	GO:0034162~toll-like receptor 9 signaling pathway	3	0.0061
	GO:0043123~positive regulation of I-kappaB kinase/NF-kappaB signaling	6	0.0092
	hsa05162:Measles	6	0.0105
	GO:0002224~toll-like receptor signaling pathway	3	0.0192
	hsa05142:Chagas disease (American trypanosomiasis)	5	0.0201
	GO:0002755~MyD88-dependent toll-like receptor signaling pathway	3	0.0280
	hsa04662:B cell receptor signaling pathway	4	0.0316
	hsa05168:Herpes simplex infection	6	0.0360

Supplementary Table S10: Mitochondrial genome sequences downloaded from Genbank for phylogenetic analyses

Specie Name	Genbank Acc Nr.	Location
C. mona **group**		
Ceropithecus mona	JQ256979.1	Sao Tome and Principe
Ceropithecus mona	JQ256980.1	Ghana
Cercopithecus campbelli	JQ256990.1	Liberia
Cercopithecus denti	JQ256988.1	-
Cercopithecus pogonias pogonias	JQ256981.1	Nigeria
Cercopithecus pogonias grayi	JQ256976.1	Republic of Congo
Cercopithecus pogonias grayi	JQ256977.1	Cameroon
Cercopithecus pogonias nigripes	JQ256978.1	Equatorial Guinea
Cercopithecus pogonias schwarzianus	JQ256989.1	Democratic Republic of Congo
Cercopithecus wolfi	JQ256984.1	Democratic Republic of Congo
Cercopithecus wolfi elegans	JQ256985.1	Democratic Republic of Congo
Cercopithecus wolfi elegans	JQ256986.1	Democratic Republic of Congo
Cercopithecus wolfi pyrogaster	JQ256987.1	Democratic Republic of Congo
C. neglectus **group**		
Cercopithecus neglectus	JQ256929.1	Uganda
C. ihoesti **group**		
Cercopithecus ihoesti	JQ256925	Democratic Republic of the Congo
Cercopithecus preussi	JQ256926	Bioko
Cercopithecus preussi	JQ256927	Bioko
Cercopithecus preussi	JQ256928	Cameroon
Cercopithecus solatus	JQ256919	Gabon
Cercopithecus solatus	JQ256920	Gabon
C. diana **group**		
Cercopithecus diana	JQ256922.1	Liberia
Cercopithecus diana	KJ434958.1	Cote d'Ivoire
Cercopithecus roloway	JQ256923.1	Ghana
C. dryas **group**		
Cercopithecus dryas	JQ256918.1	Democratic Republic of Congo
C. hamlyni **group**		
Cercopithecus hamlyni	JQ256924.1	-
C. cephus **group**		
Cercopithecus cephus cephus	JQ256947.1	Democratic Republic of Congo
Cercopithecus cephus cephus	JQ256948.1	-
Cercopithecus cephus cephus	JQ256950.1	Angola
Cercopithecus cephus cephodes	JQ256949.1	-
Cercopithecus cephus ngottoensis	JQ256939.1	Central African Republic

Cercopithecus cephus ngottoensis	JQ256948.1	Central African Republic
Cercopithecus ascanius ascanius	JQ256940.1	Democratic Republic of Congo
Cercopithecus ascanius katangae	JQ256941.1	Democratic Republic of Congo
Cercopithecus ascanius katangae	JQ256943.1	Democratic Republic of Congo
Cercopithecus ascanius schmidti	JQ256937.1	Democratic Republic of Congo
Cercopithecus ascanius schmidti	JQ256938.1	Democratic Republic of Congo
Cercopithecus ascanius whitesidei	JQ256945.1	Democratic Republic of Congo
Cercopithecus erythrogaster pococki	JQ256933.1	Nigeria
Cercopithecus erythrotis erythrotis	JQ256935.1	Bioko
Cercopithcus erythrotis camerunensis	JQ256936.1	Cameroon
Cercopithecus erythrotis camerunensis	JQ256934.1	Cameroon
Cercopithecus petaurista petaurista	JQ256931.1	Togo
Cercopithecus petaurista petaurista	JQ256983.1	Cote d'Ivoire
Cercopithecus petaurista buettikoferi	JQ256982.1	Sierra Leone
***C. nictitans* group**		
Cercopithecus nictitans nictitans	JQ256951.1	Equatorial Guinea
Cercopithecus nictitans martini	JQ256952.1	Cameroon
Cercopithecus nictitans martini (possibly museum mixup)	JQ256954.1	Democratic Republic of Congo
Cercopithecus nictitans martini (possibly museum mixup)	JQ256975.1	Democratic Republic of Congo
Cercopithecus albogularis	JQ256956.1	Zanzibar
Cercopithecus albogularis	NC_021944.1	-
Cercopithecus albogularis albotorquatus	JQ256969.1	Kenya
Cercopithecus albogularis erythrarchus	JQ256960.1	Mozambique
Cercopithecus albogularis francescae	JQ256970.1	Malawi
Cercopithecus albogularis kolbi	JQ256955.1	Kenya
Cercopithecus albogularis labiatus	JQ256961.1	South Africa
Cercopithecus albogularis moloneyi	JQ256962.1	Zambia
Cercopithecus albogularis moloneyi	JQ256971.1	United republic of Tanzania
Cercopithecus albogularis monoides	JQ256963.1	DPZ Germany
Cercopithecus doggetti	JQ256953.1	Democratic Republic of Congo
Cercopithecus doggetti	JQ256958.1	Uganda
Cercopithecus doggetti	JQ256965.1	Kenya
Cercopithecus mitis	NC_023961.1	-
Cercopithecus mitis mitis	JQ256974.1	Angola
Cercopithecus mitis boutourlinii	JQ256959.1	Ethiopia
Cercopithecus mitis heymansi	JQ256967.1	Democratic Republic of Congo
Cercopithecus mitis kandti	JQ256968.1	Democratic Republic of Congo
Cercopithecus mitis kandti	JQ256972.1	Uganda
Cercopithecus mitis opisthostictus	JQ256966.1	Zambia

Cercopithecus mitis opisthostictus	JQ256973.1	Zambia
Cercopithecus mitis stuhlmanni	JQ256957.1	Sudan
**non-*Cercopithecus* outgroups**		
Chlorocebus aethiops	NC007009.1	-
Chlorocebus sabaeus	EF597503.1	-
Chlorocebus tantalus	NC009748.1	-
Papio anubis	KJ434958,1	-
Macaca mulatta	NC005943.1	-
Rhinopithecus roxellana	KM504390.1	-
Miopithecus ogouensis	JQ256997	-
Allenopithecus nigroviridis	NC_023965	-
Erythrocebus patas	KJ434955	-
Homo sapiens	GU170820.1	-

Supplementary Table S11: Node support and divergence times for mitochondrial genome phylogenetic tree. Maximum-likelihood bootstraps values (BP), Bayesian posterior probabilities (PP), mean divergence and 95% composite credibility intervals (CI) in million years (myr). Node numbers are presented in Supplementary Figure S10

Node	BP	PP	Mean divergence	95% CI
1	100	1	33.49	28.48-38.86
2	100	1	27.94	21.97-29.92
3	100	1	16.61	13.9-18.99
4	100	1	13.64	11.59-15.87
5	100	1	9.01	7.61-10.59
6	100	1	4.24	3.53-4.48
7	100	1	11.94	9.81-13.49
8	100	1	5.18	4.41-6.11
9	100	1	2.27	1.89-2.65
10	100	1	2.47	2.07-2.88
11	100	1	10.2	8.66-11.86
12	100	1	0.96	0.78-1.14
13	100	1	9.13	7.72-10.69
14	100	1	10.03	8.38-11.60
15	100	1	4.58	3.38-5.36
16	100	1	8.19	6.79-9.45
17	100	1	3.68	3.12-4.28
18	100	1	2.78	2.34-3.21
19	100	1	1.30	1.08-1.55

Supplementary Table S12: Total length and largest block (kb) of introgressed regions in *C. mona* individuals (M10-M13) from the East clade with a posterior cutoff of 0.98 and their overlap region

ID	Total length (kb)	Largest block (kb)
M10	85,582	45
M11	83,634	43
M12	83,762	56
M13	84,949	54
**Overlap**	3,679	19

Supplementary Table S13: List of introgressed genes in overlapping regions >2kb when posterior probability ≥0.98

Contig name	Position Start	Position End	Length	Gene Name
ctg165	463000	482000	19000	*VWA5B1*
ctg15	53759000	53769000	10000	*PTPRK*
ctg7	9393000	9403000	10000	*FRAS1*
ctg42	23958000	23967000	9000	*ZC3H13*
ctg42	23958000	23967000	9000	*CPB2*
ctg8	6288000	6297000	9000	*PCSK2*
ctg35	35559000	35567000	8000	*KCNT2*
ctg42	23948000	23956000	8000	*ZC3H13*
ctg42	23948000	23956000	8000	*CPB2*
ctg67	9150000	9158000	8000	*DAPK2*
ctg28	20482000	20489000	7000	*ZRANB3*
ctg3	11339000	11346000	7000	*PTDSS1*
ctg49	13037000	13044000	7000	*FHIT*
ctg50	7768000	7775000	7000	*Null*
ctg1	44361000	44367000	6000	*DAW1*
ctg10	36230000	36236000	6000	*CNRIP1*
ctg110	9133000	9139000	6000	*EDN3*
ctg124	434000	440000	6000	*ZNF141*
ctg15	57620000	57626000	6000	*ENPP3*
ctg19	16712000	16718000	6000	*MYO16*
ctg26	10955000	10961000	6000	*SIDT1*
ctg3	11299000	11305000	6000	*PTDSS1*
ctg37	13088000	13094000	6000	*DDAH1*
ctg4	26168000	26174000	6000	*MRPS27*
ctg47	12759000	12765000	6000	*MYCBP2*
ctg58	10501000	10507000	6000	*Null*
ctg64	2593000	2599000	6000	*GRIN2B*
ctg1	11243000	11248000	5000	*GTF3C3*
ctg1	38790000	38795000	5000	*FARSB*
ctg1	44368000	44373000	5000	*DAW1*
ctg15	55243000	55248000	5000	*LAMA2*
ctg161	1840000	1845000	5000	*INPP4A*
ctg165	456000	461000	5000	*VWA5B1*
ctg2	7157000	7162000	5000	*DOCK1*
ctg21	18740000	18745000	5000	*TRGV9*
ctg29	26048000	26053000	5000	*ASAP2*
ctg3	5872000	5877000	5000	*TMEM55A*
ctg3	11347000	11352000	5000	*PTDSS1*
ctg31	4043000	4048000	5000	*F11*

ctg31	14830000	14835000	5000	*WDR17*
ctg32	10776000	10781000	5000	*TMEM232*
ctg35	35547000	35552000	5000	*KCNT2*
ctg4	23514000	23519000	5000	*FAM169A*
ctg46	1388000	1393000	5000	*DDX60L*
ctg49	13045000	13050000	5000	*FHIT*
ctg85	1666000	1671000	5000	*SCCPDH*
ctg9	19293000	19298000	5000	*Null*
ctg108	7503000	7507000	4000	*IFT122*
ctg108	8089000	8093000	4000	*ACAD9*
ctg116	8984000	8988000	4000	*MARK4*
ctg12	16902000	16906000	4000	*GRIA4*
ctg121	1836000	1840000	4000	*CCT3*
ctg128	2538000	2542000	4000	*KANK4*
ctg13	50307000	50311000	4000	*ALDH1L2*
ctg15	34346000	34350000	4000	*ZBTB24*
ctg15	53845000	53849000	4000	*PTPRK*
ctg15	55235000	55239000	4000	*LAMA2*
ctg161	1115000	1119000	4000	*TMEM131*
ctg165	1413000	1417000	4000	*CAPZB*
ctg18	16215000	16219000	4000	*B4GALT6*
ctg19	21068000	21072000	4000	*DCUN1D2*
ctg20	21745000	21749000	4000	*DOCK8*
ctg3	11333000	11337000	4000	*PTDSS1*
ctg3	13211000	13215000	4000	*POP1*
ctg30	1216000	1220000	4000	*ICA1*
ctg31	14825000	14829000	4000	*WDR17*
ctg32	11652000	11656000	4000	*STARD4*
ctg38	1912000	1916000	4000	*KRT38*
ctg4	23505000	23509000	4000	*FAM169A*
ctg42	38664000	38668000	4000	*DIAPH3*
ctg42	38664000	38668000	4000	*None*
ctg45	9646000	9650000	4000	*TRPM7*
ctg47	12791000	12795000	4000	*MYCBP2*
ctg58	8791000	8795000	4000	*CUL1*
ctg6	3469000	3473000	4000	*GABRG1*
ctg63	6501000	6505000	4000	*GUCY2F*
ctg64	4640000	4644000	4000	*PTPRO*
ctg77	2564000	2568000	4000	*SART1*
ctg8	36165000	36169000	4000	*Null*
ctg92	1231000	1235000	4000	*GRID1*
ctg1	18378000	18381000	3000	*GVQW2*

ctg1	18378000	18381000	3000	*RAPH1*
ctg1	43573000	43576000	3000	*Null*
ctg101	9639000	9642000	3000	*ANKRD27*
ctg113	297000	300000	3000	*PPP1R3E*
ctg116	8989000	8992000	3000	*MARK4*
ctg120	61000	64000	3000	*None*
ctg126	5901000	5904000	3000	*TRABD2A*
ctg13	48788000	48791000	3000	*STAB2*
ctg130	1000000	1003000	3000	*MYO18B*
ctg130	5902000	5905000	3000	*SEC14L6*
ctg131	344000	347000	3000	*MAGEC3*
ctg141	2231000	2234000	3000	*STXBP5*
ctg15	34379000	34382000	3000	*AK9*
ctg15	46629000	46632000	3000	*TBC1D32*
ctg15	46633000	46636000	3000	*TBC1D32*
ctg15	57616000	57619000	3000	*ENPP3*
ctg15	58573000	58576000	3000	*TAAR5*
ctg15	58573000	58576000	3000	*Fpr1*
ctg157	2456000	2459000	3000	*RBP3*
ctg159	2022000	2025000	3000	*ZNF367*
ctg159	2022000	2025000	3000	*GVQW2*
ctg16	10076000	10079000	3000	*C8orf34*
ctg16	11441000	11444000	3000	*Null*
ctg165	452000	455000	3000	*VWA5B1*
ctg17	23944000	23947000	3000	*TF*
ctg17	51655000	51658000	3000	
ctg18	24567000	24570000	3000	*TMEM241*
ctg20	5196000	5199000	3000	*UBQLN1*
ctg203	542000	545000	3000	*CECR2*
ctg21	12199000	12202000	3000	*NPC1L1*
ctg22	42579000	42582000	3000	
ctg225	154000	157000	3000	*LPCAT1*
ctg225	181000	184000	3000	*LPCAT1*
ctg23	3525000	3528000	3000	*ENAH*
ctg25	32672000	32675000	3000	*HMMR*
ctg25	32672000	32675000	3000	*MAT2B*
ctg26	12341000	12344000	3000	*SLC9C1*
ctg28	10750000	10753000	3000	*SCTR*
ctg28	20506000	20509000	3000	*ZRANB3*
ctg28	20859000	20862000	3000	*LCT*
ctg29	9332000	9335000	3000	*DTNB*
ctg290	105000	108000	3000	*SMARCB1*

ctg293	224000	227000	3000	*CRCP*
ctg3	11249000	11252000	3000	*MTERF3*
ctg3	11249000	11252000	3000	*UQCRB*
ctg3	23919000	23922000	3000	*None*
ctg31	4049000	4052000	3000	*F11*
ctg31	14798000	14801000	3000	*WDR17*
ctg31	14804000	14807000	3000	*WDR17*
ctg31	21298000	21301000	3000	*MFAP3L*
ctg34	11165000	11168000	3000	*PRPH*
ctg35	17762000	17765000	3000	*TOR1AIP1*
ctg4	26175000	26178000	3000	*MRPS27*
ctg42	22970000	22973000	3000	*GTF2F2*
ctg42	23968000	23971000	3000	*CPB2*
ctg42	24682000	24685000	3000	*HTR2A*
ctg45	9655000	9658000	3000	*TRPM7*
ctg46	1384000	1387000	3000	*DDX60L*
ctg5	19242000	19245000	3000	*DOCK4*
ctg57	1258000	1261000	3000	*SLC5A9*
ctg58	8782000	8785000	3000	*CUL1*
ctg6	22637000	22640000	3000	*UNC5C*
ctg6	40927000	40930000	3000	*C4orf32*
ctg6	40934000	40937000	3000	*C4orf32*
ctg62	17326000	17329000	3000	*MAP4K5*
ctg63	6485000	6488000	3000	*GUCY2F*
ctg63	6490000	6493000	3000	*GUCY2F*
ctg63	10089000	10092000	3000	*NRK*
ctg64	378000	381000	3000	*PRB1*
ctg64	378000	381000	3000	*PRB1*
ctg64	4651000	4654000	3000	*PTPRO*
ctg64	7980000	7983000	3000	*CAPZA3*
ctg64	10976000	10979000	3000	*ABCC9*
ctg7	1716000	1719000	3000	*Null*
ctg7	14467000	14470000	3000	*COPS4*
ctg7	14478000	14481000	3000	*COPS4*
ctg74	6308000	6311000	3000	*CDH12*
ctg8	197000	200000	3000	*SSTR4*
ctg85	1705000	1708000	3000	*SCCPDH*
ctg89	3180000	3183000	3000	*MYH2*
ctg9	19133000	19136000	3000	*C14orf159*
ctg9	22514000	22517000	3000	*SERPINA9*
ctg90	869000	872000	3000	*IFFO1*
ctg93	3624000	3627000	3000	*SLC9A2*

ctg94	3912000	3915000	3000	*CSMD1*
ctg94	4482000	4485000	3000	*CSMD1*
ctg95	3432000	3435000	3000	*FNBP1*
ctg1	11253000	11255000	2000	*GTF3C3*
ctg1	18402000	18404000	2000	*RAPH1*
ctg1	20525000	20527000	2000	*PARD3B*
ctg1	23794000	23796000	2000	*PTH2R*
ctg1	23798000	23800000	2000	*PTH2R*
ctg1	30870000	30872000	2000	*ABCA12*
ctg10	22212000	22214000	2000	*EML6*
ctg108	8086000	8088000	2000	*ACAD9*
ctg11	1410000	1412000	2000	*NLRP14*
ctg11	1419000	1421000	2000	*NLRP14*
ctg11	1438000	1440000	2000	*NLRP14*
ctg11	31717000	31719000	2000	*ZNF429*
ctg110	9057000	9059000	2000	*ZNF831*
ctg110	9124000	9126000	2000	*EDN3*
ctg115	2663000	2665000	2000	*MALRD1*
ctg115	6031000	6033000	2000	*PIP4K2A*
ctg116	7574000	7576000	2000	*PRKD2*
ctg116	7577000	7579000	2000	*PRKD2*
ctg12	29340000	29342000	2000	
ctg12	29340000	29342000	2000	
ctg121	1843000	1845000	2000	*CCT3*
ctg121	1857000	1859000	2000	*CCT3*
ctg121	1878000	1880000	2000	*VHLL*
ctg122	273000	275000	2000	*IDS*
ctg124	3994000	3996000	2000	*LRPAP1*
ctg129	4429000	4431000	2000	*OCA2*
ctg135	3477000	3479000	2000	*DST*
ctg139	3319000	3321000	2000	*SH3RF3*
ctg14	5677000	5679000	2000	*ABCC5*
ctg140	1583000	1585000	2000	*DIRAS2*
ctg141	2228000	2230000	2000	*STXBP5*
ctg142	1153000	1155000	2000	*ITGA8*
ctg145	314000	316000	2000	*PSD4*
ctg15	34376000	34378000	2000	*AK9*
ctg15	34385000	34387000	2000	*AK9*
ctg15	46653000	46655000	2000	*TBC1D32*
ctg15	46656000	46658000	2000	*TBC1D32*
ctg15	53787000	53789000	2000	*PTPRK*
ctg15	53818000	53820000	2000	*PTPRK*

ctg15	53821000	53823000	2000	*PTPRK*
ctg15	53939000	53941000	2000	*PTPRK*
ctg15	63128000	63130000	2000	*MAP3K5*
ctg159	2026000	2028000	2000	*ZNF367*
ctg16	11393000	11395000	2000	*ARFGEF1*
ctg16	11462000	11464000	2000	*Null*
ctg16	11542000	11544000	2000	*COPS5*
ctg16	12560000	12562000	2000	*DNAJC5B*
ctg17	31924000	31926000	2000	*PXYLP1*
ctg17	47277000	47279000	2000	*PLCH1*
ctg18	13356000	13358000	2000	*Null*
ctg18	16229000	16231000	2000	*B4GALT6*
ctg19	3090000	3092000	2000	*DNAJC3*
ctg19	7915000	7917000	2000	*PCCA*
ctg19	16721000	16723000	2000	*MYO16*
ctg19	21337000	21339000	2000	
ctg2	7144000	7146000	2000	*DOCK1*
ctg2	12236000	12238000	2000	*BTBD16*
ctg2	12236000	12238000	2000	*TACC2*
ctg2	30118000	30120000	2000	*SORCS3*
ctg2	30121000	30123000	2000	*SORCS3*
ctg2	48991000	48993000	2000	*SGMS1*
ctg2	50206000	50208000	2000	*PRKG1*
ctg20	4511000	4513000	2000	*SLC28A3*
ctg20	5189000	5191000	2000	*UBQLN1*
ctg20	5207000	5209000	2000	*UBQLN1*
ctg20	5210000	5212000	2000	*UBQLN1*
ctg21	19932000	19934000	2000	*ELMO1*
ctg22	34976000	34978000	2000	*METTL8*
ctg22	37799000	37801000	2000	*OLA1*
ctg22	37802000	37804000	2000	*OLA1*
ctg22	37876000	37878000	2000	*OLA1*
ctg22	37876000	37878000	2000	*Null*
ctg225	454000	456000	2000	*SLC6A19*
ctg225	454000	456000	2000	*SLC6A18*
ctg23	11105000	11107000	2000	*TGFB2*
ctg23	13022000	13024000	2000	*ESRRG*
ctg24	24172000	24174000	2000	*BNC2*
ctg240	436000	438000	2000	*SLC28A1*
ctg25	14686000	14688000	2000	*TCERG1*
ctg26	23771000	23773000	2000	*PCNP*
ctg275	97000	99000	2000	*P2RX4*

ctg28	20726000	20728000	2000	*R3HDM1*
ctg29	9336000	9338000	2000	*DTNB*
ctg293	228000	230000	2000	*CRCP*
ctg3	12105000	12107000	2000	*CPQ*
ctg3	13199000	13201000	2000	*POP1*
ctg3	13208000	13210000	2000	*POP1*
ctg3	49352000	49354000	2000	*KCNQ3*
ctg3	49356000	49358000	2000	*KCNQ3*
ctg30	18118000	18120000	2000	*STK31*
ctg32	12442000	12444000	2000	*EPB41L4A*
ctg34	7197000	7199000	2000	*ANO6*
ctg342	92000	94000	2000	*UGT2A3*
ctg35	5573000	5575000	2000	*TIPRL*
ctg35	35543000	35545000	2000	*KCNT2*
ctg36	9843000	9845000	2000	*SNX29*
ctg37	13085000	13087000	2000	*DDAH1*
ctg4	26130000	26132000	2000	*MRPS27*
ctg42	13345000	13347000	2000	*NBEA*
ctg44	14942000	14944000	2000	*COL12A1*
ctg44	17293000	17295000	2000	*KCNQ5*
ctg45	1632000	1634000	2000	*AQP9*
ctg45	9652000	9654000	2000	*TRPM7*
ctg45	9671000	9673000	2000	*TRPM7*
ctg46	13367000	13369000	2000	*GLRB*
ctg47	12739000	12741000	2000	*MYCBP2*
ctg48	10157000	10159000	2000	*PLPPR5*
ctg48	15114000	15116000	2000	
ctg49	20808000	20810000	2000	*TKT*
ctg49	24436000	24438000	2000	*TRAIP*
ctg49	24446000	24448000	2000	*TRAIP*
ctg49	24436000	24438000	2000	*UBA7*
ctg49	24446000	24448000	2000	*UBA7*
ctg49	24436000	24438000	2000	*UBA7*
ctg49	24446000	24448000	2000	*UBA7*
ctg5	10366000	10368000	2000	*Null*
ctg55	3051000	3053000	2000	*RNF20*
ctg56	17911000	17913000	2000	*GEMIN8*
ctg56	17914000	17916000	2000	*GEMIN8*
ctg57	1721000	1723000	2000	*AGBL4*
ctg59	7999000	8001000	2000	*EXOC7*
ctg6	18093000	18095000	2000	*CCSER1*
ctg6	40931000	40933000	2000	*C4orf32*

ctg6	47423000	47425000	2000	*PRSS12*
ctg6	79176000	79178000	2000	*NR3C2*
ctg60	5039000	5041000	2000	*TAF12*
ctg60	5052000	5054000	2000	*TAF12*
ctg61	11118000	11120000	2000	*GINS4*
ctg62	17309000	17311000	2000	*MAP4K5*
ctg62	17315000	17317000	2000	*MAP4K5*
ctg62	17320000	17322000	2000	*MAP4K5*
ctg63	10086000	10088000	2000	*NRK*
ctg64	823000	825000	2000	*ETV6*
ctg64	10973000	10975000	2000	*ABCC9*
ctg67	6212000	6214000	2000	*SMAD6*
ctg68	3515000	3517000	2000	
ctg68	13115000	13117000	2000	*MEGF6*
ctg7	9350000	9352000	2000	*FRAS1*
ctg7	14185000	14187000	2000	*SCD5*
ctg74	6323000	6325000	2000	*CDH12*
ctg77	2557000	2559000	2000	*SART1*
ctg77	2569000	2571000	2000	*SART1*
ctg8	29029000	29031000	2000	*PHF20*
ctg82	10811000	10813000	2000	*OR56A5*
ctg82	10829000	10831000	2000	*OR51A4*
ctg84	8409000	8411000	2000	*EVA1C*
ctg85	1693000	1695000	2000	*SCCPDH*
ctg85	1696000	1698000	2000	*SCCPDH*
ctg85	1777000	1779000	2000	*CNST*
ctg86	10669000	10671000	2000	*Null*
ctg89	3184000	3186000	2000	*MYH2*
ctg9	18681000	18683000	2000	*TTC7B*
ctg9	19130000	19132000	2000	*C14orf159*
ctg9	19310000	19312000	2000	*Null*
ctg9	22139000	22141000	2000	*IFI27*
ctg92	1326000	1328000	2000	*GRID1*
ctg92	5036000	5038000	2000	*NRG3*
ctg94	790000	792000	2000	*DLGAP2*
ctg96	470000	472000	2000	*NOTCH3*
ctg96	12798000	12800000	2000	*NCLN*
